# Addressing the Challenges of Solid-State Nanopores: Strategies for Performance Enhancement

**DOI:** 10.3390/ijms27062536

**Published:** 2026-03-10

**Authors:** Xi Chen, Jiayi Liu, Zhiyou Xiao, Guowei Wang, Yu Li, Hongwen Wu, Derong Xu

**Affiliations:** 1 Jiangxi Institute of Translational Medicine, Jiangxi Medical College, Nanchang 330006, China; chenxi@email.ncu.edu.cn; 2 First Clinical Medical College, Jiangxi Medical College, Nanchang University, Nanchang 330006, China; 3 National Engineering Research Center for Bioengineering Drugs and the Technologies, Institute of Translational Medicine, Jiangxi Medical College, Nanchang University, Nanchang 330031, China; 18838106951@163.com (J.L.); 15070976268@163.com (Z.X.); 15949609587@163.com (G.W.); 19921870092@163.com (Y.L.); 4 Huankui Academy, Nanchang University, Nanchang 330031, China; 5 Jiangxi Provincial Key Laboratory of Prevention and Treatment of Infectious Diseases, Jiangxi Medical College, Nanchang University, Nanchang 330052, China; ndyfy04710@ncu.edu.cn

**Keywords:** solid-state nanopores, DNA sequencing, noise reduction, surface functionalization, translocation speed control, nanopore clogging, CMOS, CNP, machine learning

## Abstract

Solid-state nanopore sequencing, a key third-generation sequencing technology, offers considerable potential for genomics and diagnostics due to its long read lengths, real-time detection, and amplification-free operation. The technology identifies DNA sequences by measuring characteristic changes in ionic current as single-stranded DNA translocates through a nanoscale pore. However, its practical development faces challenges including limited spatiotemporal resolution, pore clogging from nonspecific adsorption, and significant electrical noise. This review systematically examines strategies developed to address these limitations. We discuss the use of ultrathin two-dimensional materials such as graphene and molybdenum disulfide to improve spatial resolution, and methods to modulate DNA translocation through optimized solution conditions, pore geometry, surface charge engineering, and bio-solid hybrid pore designs. Furthermore, we detail noise suppression strategies targeting key sources like thermal noise, 1/f noise, and dielectric noise. These approaches encompass careful material selection, surface coatings, innovations in chip and amplifier design, and machine learning–based signal processing. The review also outlines surface functionalization techniques that reduce clogging and enhance analytical specificity. While challenges remain, continued convergence of materials science, nanofabrication, and data science is advancing solid-state nanopore technology toward reliable, high-precision sequencing platforms, promising to significantly impact personalized medicine and biological research.

## 1. Introduction

With the arrival of the precision medicine era [1], sequencing based methods such as genetic disease diagnosis, tumor gene profiling, genomic research, and microbial detection [2,3,4,5], have become increasingly common. This growing adoption places greater demands on sequencing technology while also accelerating the development of diverse sequencing platforms. In 1977, Frederick Sanger revolutionized the field by introducing the dideoxy chain-termination method, which laid the critical foundation for large-scale DNA sequencing [6]. This approach utilizes dideoxy nucleotides to terminate DNA synthesis, producing fragments of varying lengths that are then separated via electrophoresis to determine the base sequence. Although Sanger sequencing opened the door to DNA reading, its high cost, short read length, and limited accuracy restricted widespread adoption.

Next-generation sequencing (NGS) operates on the principle of fragmenting DNA, immobilizing it on a solid surface, and amplifying it through bridge PCR to form clusters. Sequencing is performed by cyclic synthesis, where fluorescently labeled dNTPs are added sequentially, and signals are captured to enable massively parallel sequencing. However, NGS is constrained by short read lengths, potential PCR bias and errors, complex data analysis, and high costs [7,8].

**Figure 1:**
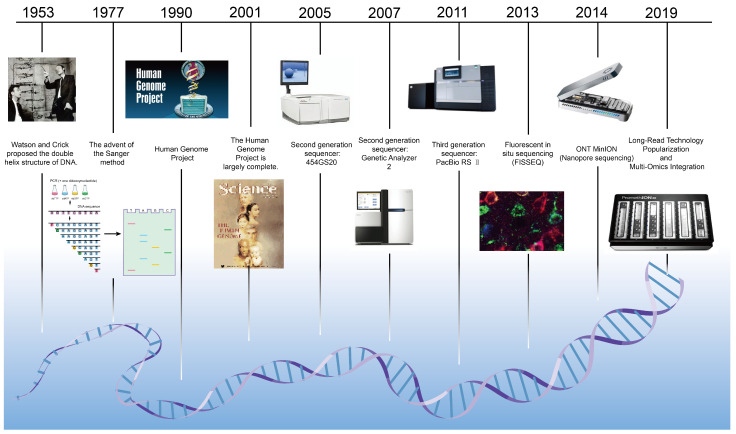
The Evolution of Sequencing Technologies.

The emergence of third-generation sequencing (TGS) in 2011 marked another leap forward [8,9], with two major platforms leading the field: single-molecule real-time sequencing developed by Pacific Biosciences (PacBio) [10], and nanopore sequencing pioneered by Oxford Nanopore Technologies (ONT) [10,11]. Among these, solid-state nanopores fabricated from insulating thin films exhibit superior mechanical and chemical stability, along with tunable pore dimensions and geometry [12]. These features provide advantages such as low cost, stable performance, and reusability, highlighting their promising potential in genomics, transcriptomics, and medical diagnostics [13,14]. Figure 1 provides a brief overview of the development of DNA sequencing.

## 2. Principle

**Figure 2:**
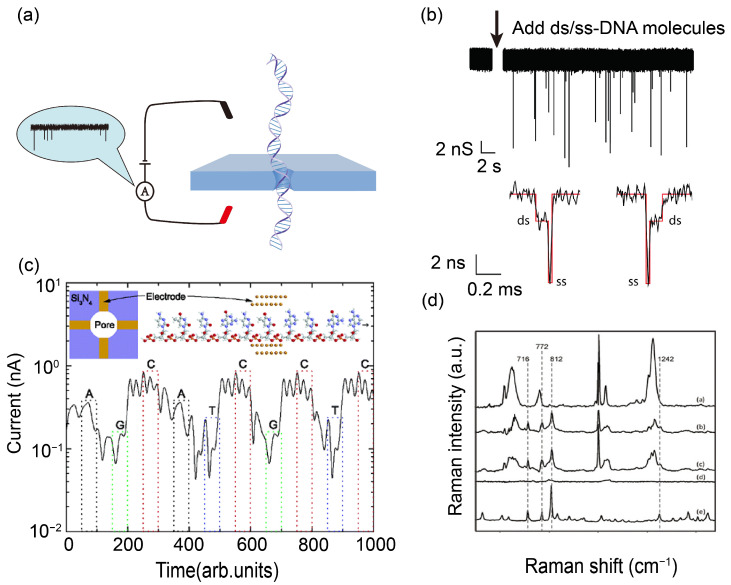
Detect various signals in DNA. (**a**) Schematic diagram of a typical solid-state nanopore device for ion current detection experiments. (**b**) DNA characteristic signals obtained by detecting ionic currents, along with typical time-course profiles of DNA addition before and after solution addition and pore conductivity. DNA pass-through is distinguished based on the characteristics of the blocking current: the double-stranded ds portion enters the nanopore first (left), while the single-stranded ss portion enters first (right). Reproduced from ref. [19]. © 2010 American Chemical Society. (**c**) Different bases are characterized by detecting transverse current. Reproduced from ref. [21]. © 2006 American Chemical Society. (**d**) Detecting different DNA sequences using Raman spectroscopy. Reproduced from ref. [23]. © 2025 American Chemical Society.

The working principle of solid state nanopore sequencing is simple. As shown in Figure 2a, the device includes a nanopore, which is a tiny aperture with a diameter between 1 and 100 nm, placed between two electrodes [15]. Under an applied electric field, molecules passing through the nanopore induce measurable changes in various signals, such as ionic current (Figure 2b) [16,17,18,19], transverse current (Figure 2c) [18,20,21,22], Raman spectroscopy profiles (Figure 2d) [23], and other detectable signals [24,25]. Detecting ion currents is currently the most widely used method. enabling direct sequencing by monitoring electrical signal changes as individual DNA molecules traverse nanoscale channels [26,27]. As a representative of third-generation sequencing, it has attracted considerable attention for its potential to deliver long read lengths, high throughput, and amplification-free operation [12,13,14]. The key component of this platform is a nanopore fabricated in an ultrathin solid-state membrane, typically made of silicon nitride or graphene. The system is immersed in an electrolyte solution, and a voltage applied across the pore generates a stable ionic current known as the “open-pore current.” When a DNA molecule translocates through the pore in an extended linear conformation under the applied voltage, its physical volume and constituent bases temporarily occupy the pore space. This displaces electrolyte ions within the nanopore, causing an instantaneous drop in ionic current and generating a characteristic “blocking signal”.

Sequencing is possible because the four DNA bases, adenine (A), thymine (T), cytosine (C), and guanine (G), each disrupt the ionic current in a distinct way. These differences arise from their unique chemical structures, sizes, and charge distributions. For example, the larger purine bases (A and G) typically cause deeper current blockades than the smaller pyrimidine bases (C and T). As a DNA strand moves through the pore at a controlled speed, highly sensitive instruments record the corresponding current changes in real time. This produces a characteristic profile of current over time. Finally, advanced machine learning and pattern recognition algorithms compare these brief current patterns against known signatures for each base. This process converts the electrical data into the final DNA sequence, which is the exact order of the A, T, C, and G bases [17,28].

While nanopores theoretically offer single-base resolution [14], their practical performance often falls below expectations due to several limiting factors. These include, but are not limited to, insufficient spatial and temporal resolution, suboptimal signal-to-noise ratios, and specificity that requires further improvement [14,26,29,30,31,32]. As a result, the broader adoption and scalability of nanopore technology remain constrained.

In this review, we provide a concise introduction to the principles and methods of nanopore sequencing, with a focus on current challenges. We summarize potential solutions based on prior research and conclude with a retrospective analysis of the development of solid-state nanopore sequencing, along with a forward-looking perspective on its future. We hope this work will help address some of the experimental challenges faced by researchers in the field and inspire the development of new functionalities for nanopore-based platforms.

## 3. Drawbacks and Resolution

### 3.1. Low Spatial Resolution

Among the various materials employed in fabricating solid-state nanopores, silicon nitride (SiN_x_) is the most widely used. Nanopores fabricated in insulating thin films such as SiN_x_ exhibit excellent mechanical and chemical stability, along with greater flexibility in controlling pore size and geometry [12,33]. However, conventional solid state membranes are usually more than 10 nm thick. To distinguish individual DNA bases, the membrane should ideally be thinner than 0.5 nm. The optimal thickness would be close to the length of a single DNA base pair, which is about 0.34 nm [30,34,35,36]. As a result, nanopores formed in standard silicon nitride films possess sensing regions that are too long to resolve individual nucleotides effectively. This fundamental limitation has motivated extensive research into alternative materials and fabrication techniques capable of producing ultrathin solid-state membranes.

**Figure 3:**
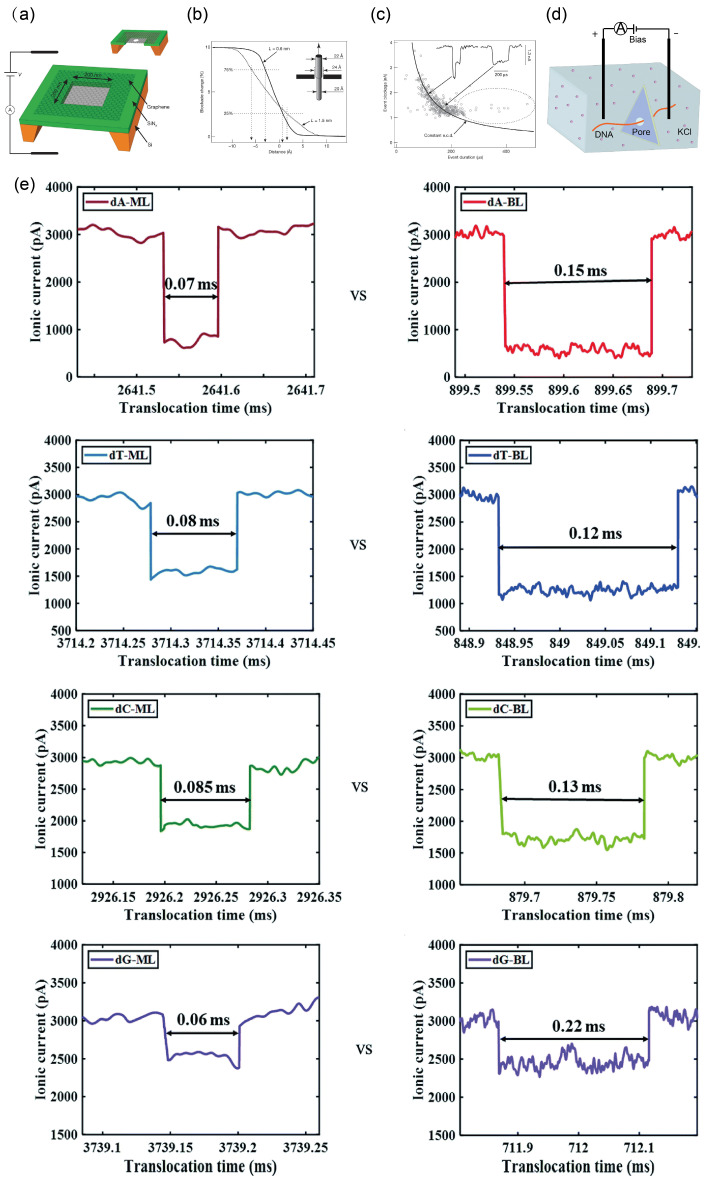

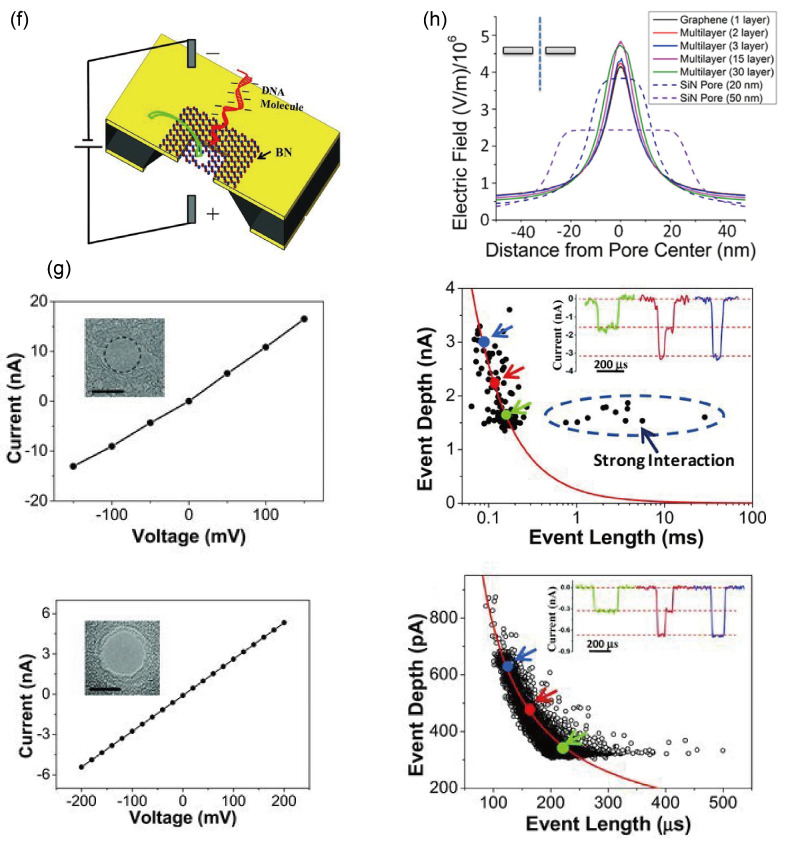
Enhancing the Time Resolution of Nanopores Using Ultrathin Materials. (**a**) A graphene membrane was mounted over a 200  ×  200 nm aperture in SiN_x_ suspended across a Si frame (not to scale). (**b**) The spatial resolution of nanopores with insulation thicknesses of 1.5 nm and 0.6 nm is 7.5 Å and 3.5 Å, respectively. (**c**) The inset in the upper right shows pulse signals generated by passing through nanopores in folded (left) and linear (right) configurations; the hyperbolic curves correspond to free translocation events under fixed e.c.d. (electronic charge deficit) conditions. Encircled events are delayed by graphene–DNA interactions. Panels (**a**–**c**) are reproduced with permission from ref. [37]. © 2010 Springer Nature. (**d**) Schematic diagram of a bilayer MoS_2_ nanopore device. (**e**) Using MoS_2_ nanopores to detect poly A, poly T, poly C, and poly G, bilayer MoS_2_ nanopores extend DNA translocation time compared to monolayer MoS_2_ nanopores. Panels (**d**,**e**) are reproduced with permission from ref. [42]. © 2021 Royal Society of Chemistry. (**f**) Schematic of BN nanopore device. (**g**) I-V curves of BN nanopore (top) and SiN nanopore (bottom). Insets: Pulse signals generated by translocation of fully folded (left), partially folded (middle), and linear (right) DNA. The events indicated by arrows of different colors are consistent with the specific pulse images of the same color in the insets. Panels (**f**,**g**) are reproduced with permission from ref. [43]. © 2013 WILEY-VCH. (**h**) Electric field distribution along the axis of the pore with varying. Reproduced from ref. [52]. © 2013 American Chemical Society.

In 2010, Golovchenko and colleagues used a 0.5 × 0.5 mm piece of chemical-vapor-deposited (CVD) graphene mounted on an independent, insulating SiN_x_ chip containing a 200 × 200 nm window and a 250 nm-thick frame [37]. A nanopore was then etched into the graphene using a focused electron beam (Figure 3a). Similar to classical solid-state nanopore setups, the graphene membrane separated two ionic chambers. Silver/silver-chloride electrodes were placed in the electrolyte reservoirs on both sides and connected via wires to a power source and sensors. By solving the Laplace equation numerically for the ionic current density under appropriate conductivity and boundary conditions, and integrating over the pore region, the resulting conductivity was found to be equivalent to solving the Poisson-Nernst-Planck equation in the same geometry. The model yielded an effective graphene insulating thickness of 0.6 nm (+0.9, −0.6 nm).

Golovchenko et al. further explored the spatial resolution limits of graphene films through a physical experiment [37]. They modeled a long insulating cylinder 2.2 nm in diameter passing symmetrically through a nanopore 2.4 nm in diameter. Along its length, the cylinder’s diameter abruptly changed from 2.2 nm to 2.0 nm. Calculations of ionic conductivity showed that, with an insulating layer thickness of 0.6 nm, the nanopore’s spatial resolution could reach 3.5 Å at the point of maximum blockade change (25% to 75%; Figure 3b). More notably, the authors used the same graphene membrane to detect DNA molecules and successfully distinguished between different translocation morphologies, including linear and folded configurations (Figure 3c).

Owing to the strong hydrophobicity of both DNA and graphene, DNA molecules frequently adhere to graphene nanopores during sequencing, leading to pore blockage and hindering reliable DNA detection [34,38,39]. This adhesion reduces the practical efficiency and throughput of graphene-based devices. Furthermore, the atomically thin graphene membrane exhibits significant mechanical undulations, resulting in elevated electrical noise during ionic current measurements [37]. Molybdenum disulfide (MoS_2_) has become a promising alternative to graphene. It can achieve similar atomic-scale thinness while being less hydrophobic. In 2013, Amir et al. used hafnium oxide (HfO_2_) as a gate insulator to increase the room-temperature mobility of monolayer MoS_2_ by about 200 times, a gain that exceeded what was observed in graphene [40]. Further advances include the 2018 work by Luan et al., who achieved spontaneous DNA translocation using chemical potential rather than electrical bias [41], and Payel’s 2021 experiments showing that charge interactions in bilayer MoS_2_ nanopores can slow DNA translocation (Figure 3d,e) [42]. Together, these studies illustrate the progressive refinement of MoS_2_ as a promising material for solid-state nanopore sequencing.

Boron nitride (BN), a III–V compound often referred to as “white graphene”, possesses a crystal structure analogous to graphene [43]. It consists of a two-dimensional (2D) honeycomb lattice of alternating boron and nitrogen atoms bound through sp^2^ hybridization. BN shares many of graphene’s advantageous properties, including high-temperature stability, excellent electrical insulation, high mechanical strength, large thermal conductivity, considerable hardness, and strong corrosion resistance [44,45]. Moreover, the thickness of monolayer BN (~0.33 nm) is comparable to the inter-nucleotide spacing in single-stranded DNA (0.32–0.52 nm), making it a competitive candidate for achieving single-base resolution on ultrathin nanopore platforms [43,46,47]. Compared with graphene, BN exhibits inherent hydrophilicity, lower electrical noise, and chemically inert edges, which collectively reduce DNA adsorption and signal interference [47,48,49]. Relative to molybdenum disulfide, its uniform atomic structure and absence of charge defects contribute to more stable ionic currents and improved signal-to-noise ratios, offering an ideal platform for high-precision, long-lifetime biosensing [50].

Liu et al. [43] first experimentally demonstrated DNA translocation through boron-nitride nanopores. They grew hexagonal BN films on copper foil via chemical vapor deposition (CVD) and then drilled nanopores of various sizes into suspended BN membranes using a 300 kV electron beam (a process completed in under 2 s). The BN membrane separated cis and trans chambers, allowing ion passage only through the nanopores. A bias voltage applied across the membrane drove DNA molecules through the pores. The chip was sealed between two polyetheretherketone (PEEK) chambers with a polydimethylsiloxane (PDMS) gasket; both chambers were filled with KCl solution and connected to Ag/AgCl electrodes. Nanopore conductance was measured in 3 M KCl (pH 10, 10 mM Tris, 1 mM EDTA), with the experimental setup shown in Figure 3f. BN nanopores showed a conductance of approximately 100 nS, which was notably higher than the 26.07 nS measured for SiN nanopores of similar size. This difference is attributed to the thinner nature of BN.

As shown in Figure 3g, Liu et al. observed three characteristic translocation signals in DNA experiments, identical to those seen in conventional SiN nanopores: unfolded (linear), partially folded, and fully folded conformations, with equivalent event-charge-deficit (ECD) behavior [37]. Similar to graphene nanopores, longer translocation events were recorded, indicating strong interactions between the hydrophobic BN surface and DNA molecules. This finding informs later discussions regarding slowed DNA translocation [37,47]. Notably, the authors employed an unconventional approach to verify nanopore thickness and diameter: using a Poisson–Boltzmann formulation [51] and assuming a DNA radius of 1.25 nm, they plotted pore-geometry-variation curves for both open-pore and DNA-blocked currents. This calculation yielded a thickness of 1.1 nm and a diameter of 4.7 nm, values consistent with transmission electron microscopy (TEM) measurements [27].

As scientists delve deeper into ultrathin materials, they have discovered that achieving single-base detection is far from being solved merely by reducing nanopore thickness to 0.34 nm. The primary obstacle lies in access resistance. Access Resistance refers to the additional resistance generated when ions converge from the bulk solution into the nanopore entrance due to streamline constriction. Using the resistance expressions $R_{pore}=\frac{\rho l}{\pi r^{2}}$ and $R_{access}=\frac{\rho }{\pi r}$, where ρ is the solution resistivity, l is the length of the pore, and *r* is the radius of the pore [52], the increased contribution of access resistance can be quantified. For a membrane with a pore size of 2 nm, the access resistance of a 50 nm thick membrane accounts for only 3.0% of the total resistance. In contrast, a 0.3 nm thick membrane exhibits a significantly larger access resistance contribution of 83.9% [52]. Freedman et al. measured the electric field across graphene films of varying layer numbers. Their results indicate that the electric field in a monolayer graphene film is nearly identical to that in a 30-layer film (Figure 3h). This suggests that beyond a certain thickness reduction, pursuing thinner nanopores offers little further benefit. Garaj et al.’s experiments corroborate this: even in single-layer graphene nanopores tightly matched to DNA, the intrinsic resolution along the molecular length is approximately 0.6 nm, failing to achieve single-base spacing [53]. In summary, once nanopore thickness is reduced to a certain level, the primary challenge shifts from thickness to access resistance. Efforts should focus on enhancing electric field focusing while maintaining a sufficient pore thickness. Freedman et al. propose that an ideal nanopore thickness should be half the aperture diameter to achieve strong electric field focusing [52]. Wang et al. pursued a manufacturing solution: they employed a high-dielectric-constant coating (Al_2_O_3_) and an asymmetric pore geometry (conical base and short straight tip) [54]. Under this design, electric field lines “leak” from the pore into the high-dielectric layer, forming a strong normal electric field at the pore edge. This actively reconfigures the distribution of the electric field in the access resistance region. As $R_{access}=\frac{\rho }{\pi r}$ indicates, access resistance is proportional to the solution’s resistivity ρ. Creating a salt concentration gradient across the pore generates a localized electrochemical potential well at the aperture, effectively compressing the spatial decay length of the electric field [55]. Only by simultaneously addressing nanopore thickness and access resistance can the goal of single-base resolution be approached.

While the previous sections have focused on optimizing the initial spatial resolution through material selection and pore geometry, the practical translation of solid-state nanopores from laboratory demonstrations to routine applications depends critically on two factors that extend beyond the detection event itself: the long-term stability of the nanopore under operational conditions, and the reliable delivery of intact, long DNA molecules to the pore.

A fundamental assumption in most nanopore experiments is that the pore geometry remains constant throughout the measurement. However, under the high electric fields (typically 10^5^–10^6^ V/cm) and variable pH conditions used to drive DNA translocation, the pore edges can undergo gradual degradation, leading to “pore expansion” over time. This phenomenon directly compromises spatial resolution, as an enlarging pore increases the effective sensing length and reduces the distinction between adjacent bases.

For BN nanopores, the edges can be either B-terminated or N-terminated, each with different chemical reactivity. Under high electric fields in aqueous electrolytes, hydrolysis reactions can occur at the pore edges, slowly etching the material and increasing the pore diameter [56]. This gradual increase in baseline conductance during an experiment compromises reproducibility and can lead to loss of the very spatial resolution that ultrathin materials promise.

For MoS_2_ nanopores, the issue is somewhat different. While MoS_2_ offers excellent electronic properties, its edges and defects are known catalytic sites for hydrogen evolution reactions (HER) [57]. Under negative bias in aqueous solutions, this reactivity can lead to electrochemical erosion, inducing thinning of the membrane around the pore or the formation of nanobubbles. These changes alter the effective pore geometry, increase noise, and degrade spatial resolution over time.

Even for the workhorse material SiN_x_, pore expansion is not negligible. Under high bias, local Joule heating and electrochemical reactions can slowly oxidize or etch the material, leading to a gradual increase in pore size [58]. This is often managed by limiting voltage or using protective coatings (e.g., ALD-deposited HfO_2_ or TiO_2_) that passivate the pore surface and enhance chemical inertness [40,59].

The second challenge that directly impacts achievable spatial resolution is the reliable delivery of intact, long DNA molecules to the nanopore. The promise of nanopore sequencing lies in its ability to read extremely long fragments (tens to hundreds of kilobases), providing crucial information for genome assembly and structural variant detection. However, this advantage is severely undermined if the DNA is sheared into smaller fragments during sample preparation or delivery.

DNA shearing occurs primarily due to hydrodynamic forces during pipetting, vortexing, or flow through microfluidic channels. Long DNA molecules (>50 kb) are particularly susceptible to breakage under even modest shear forces. Standard laboratory practices—such as pipetting with narrow-bore tips, vortexing, or excessive freeze–thaw cycles—can dramatically reduce the average fragment length, effectively negating the long-read advantage and forcing the system to resolve shorter fragments where the benefits of ultrathin membranes are less pronounced. To preserve DNA integrity and fully leverage the spatial resolution of advanced nanopores, specialized handling protocols are required: (1) Using wide-bore pipette tips and avoiding rapid aspiration or dispensing; (2) designing microfluidic channels with large cross-sections and low flow velocities; (3) using electric fields rather than pressure-driven flow to move DNA, reducing hydrodynamic shear [60]; (4) embedding long DNA in protective matrices and releasing it only near the pore.

Beyond shearing, delivery efficiency to the nanoscale pore itself is a challenge. The capture radius of a nanopore is limited (on the order of the pore diameter), meaning that DNA must diffuse or be driven very close to the pore to be captured. For long molecules, their large hydrodynamic radius and tendency to entangle further reduce capture rates. Strategies to enhance delivery include salt gradients for electrokinetic focusing [61] and integrated microfluidic channels to guide molecules toward the sensing region [62]. In summary, achieving single-base resolution requires not only optimizing the initial pore geometry but also ensuring that this geometry remains stable throughout the measurement and that intact, long DNA molecules can be reliably delivered to the sensing zone. These “upstream” challenges are as critical to spatial resolution as the material choices and fabrication techniques discussed earlier.

### 3.2. Low Temporal Resolution

The foundation of nanopore sequencing lies in detecting the characteristic electrical blockade signals generated when DNA strands translocate through a nanopore, with each base or base combination producing a distinct electrical “fingerprint” [17,63,64,65]. Ideally, the passage of each nucleotide through a solid-state nanopore would yield a clear, discrete, and uniquely identifiable current pulse [26,66,67,68,69]. In practice, however, DNA translocates at extremely high speeds, typically on the order of 1 to tens of bases per microsecond [26,65,70,71]. Meanwhile, the measurement system is usually limited to a bandwidth of about 1 MHz due to amplifier constraints and capacitive noise. This bandwidth corresponds to a reliable temporal resolution on the microsecond scale, whereas the residence time of a nucleotide within the pore ideally should be at least 10–1000 µs to permit clear signal discrimination [71,72]. As a result, extremely rapid translocation events exceed the system’s response capability, causing signal attenuation and loss of fine detail [72,73,74]. Consequently, signals from multiple bases often overlap and merge into a prolonged, complex composite pulse [70,75], analogous to capturing a fast-moving object with a slow shutter speed: the resulting “blurred” signal obscures individual features and reduces its practical utility [73,75,76].

Improving time resolution can be achieved by reducing the translocation speed of DNA or increasing bandwidth. However, according to fundamental principles of electronics, the noise power of a system is proportional to its bandwidth. Consequently, higher bandwidth introduces more noise, potentially overwhelming the already faint blockage signal [75,77]. This creates a challenging trade-off between bandwidth and signal-to-noise ratio (SNR) [72,75,76,77]. Against the backdrop of ever-increasing bandwidth, mitigating the accompanying high noise levels presents a significant challenge. A detailed discussion of noise is provided below. Moreover, even with currently available high-bandwidth systems (10 MHz), the detection time limit remains >100 ms, whereas the translocation time for a single base is significantly shorter [78]. Therefore, to achieve single-base resolution, efforts must focus on both increasing bandwidth and reducing DNA translocation speed. Here, we primarily discuss enhancing temporal resolution by decreasing DNA translocation speed.

#### 3.2.1. Regulation of Physical Conditions and Solution Properties

Over the past two decades, extensive research has investigated how various parameters influence DNA translocation. These include applied voltage [31,63,79,80,81,82,83,84,85], temperature [86], salt concentration [81,87], and solution viscosity [80,82]. For instance, simply lowering the applied voltage reduces the electrophoretic force on DNA, thereby slowing its translocation [17,85,88,89]. Adding viscous agents such as glycerol or polyethylene glycol to the buffer increases hydrodynamic resistance and decelerates the process [31,85]. Creating a salt concentration gradient across the membrane, such as high salt on the cis side and low salt on the trans side, generates an electroosmotic flow that opposes the electrophoretic force on DNA. Under carefully chosen gradient conditions, these two forces can nearly balance, significantly slowing or even temporarily trapping DNA molecules.

DNA traversing nanopores is typically driven by propulsive forces (primarily electric field forces) [65] and hindered by resistive/random forces (such as viscous resistance, electroosmotic flow, interactions with the pore channel, and Brownian motion) [66]. The net resultant force propels DNA through the nanopore. Assuming dsDNA molecules can only move along the nanopore’s symmetry axis and neglecting end-effect influences, the displacement velocity of dsDNA driven by the electric field through the pore can be determined by solving a coupled system of equations: the Stokes equation for flow between DNA and the pore surface, and the Poisson equation for the electric potential distribution within the pore [90,91]. With non-slip boundary conditions for flow and with known z-potentials on DNA and pore surfaces, the translocation velocity of the dsDNA molecule is:

$$v=\varepsilon \frac{\xi_{d}-\xi_{w}}{\eta }E$$

where ε is the dielectric constant of the electrolyte, η the viscosity of the electrolyte, $\xi_{d}$ and $\xi_{w}$ the zeta potentials on DNA and pore surfaces respectively, and *E* the biasing electric field [89].

Fologea et al. [92] noted that a single molecule within a nanopore can induce a detectable ionic blockage current (transition time), with the transition time depending on solution conditions (ionic concentration, viscosity, and temperature), nanopore properties, applied bias, and the passing molecule. Using a simple force balance equation between electrodynamic forces within the nanopore and viscous resistance across the entire molecule, the transition time is inferred as:

$$t_{d}=K\frac{\eta L_{DNA}}{\lambda V}$$

where η is the viscosity of the solution, λ and L_DNA_ are the linear charge density and length of the DNA molecule, respectively, and K is a constant of proportionality accounting for complex issues beyond the capabilities of the simple model.

**Figure 4:**
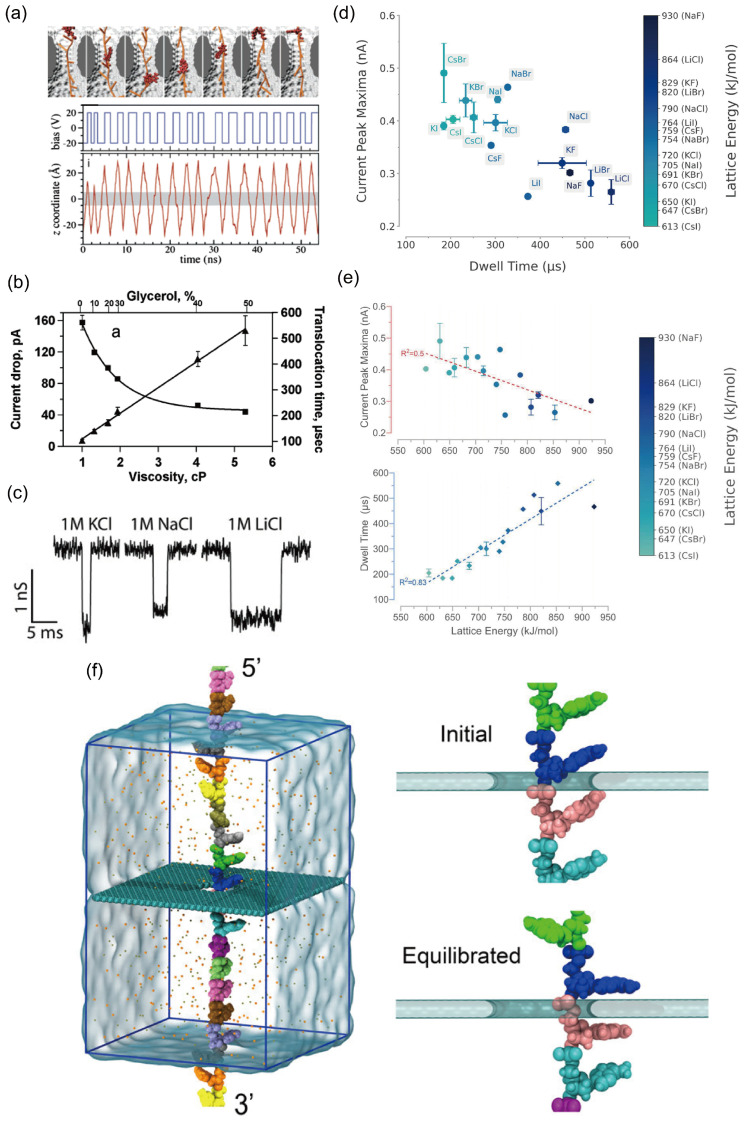
(**a**) By periodically altering the direction of the bias electric field, DNA is made to move back and forth through the nanopore. Reproduced from ref. [77]. © The Royal Society of Chemistry 2012. (**b**) Current blockage (■) and translocation time (▲) versus viscosity for 3 kbp DNA in 1.5 M KCl-TE solution at 120 mV bias voltage. The solid curves are fits for ∆Ib ∼ 1/η and td ∼ η. Reproduced from ref. [80]. © 2005 American Chemical Society. (**c**) DNA translocation events in electrolytes containing 1 M KCl, NaCl, and LiCl. Reproduced from ref. [68]. © 2012 American Chemical Society. (**d**) Effects of different alkali metal halide salts on translocation event signals in 4.8 kb linear dsDNA. This figure displays average current peaks and residence times, with each salt color-coded according to its associated lattice energy. (**e**) Linear regression plots of current and residence time versus lattice energy. Panels (**d**,**e**) are reproduced with permission from ref. [28]. CC-BY 4.0. (**f**) Molecular dynamics simulation of stretched poly(dA) ssDNA being threaded through a graphene nanopore. Reproduced from ref. [100]. © 2015 American Chemical Society.

It is readily understood that reducing the electric field driving DNA movement can slow the translocation speed, thereby prolonging the time each DNA molecule spends inside the nanopore. This extended dwell time allows more detailed information to be collected from the DNA as it passes through, which is crucial for applications in DNA and protein detection using nanopores. However, simply lowering the bias voltage is not a straightforward solution. On one hand, it reduces the number of molecules processed per unit time [34], which conflicts with the high-throughput advantage of nanopore sequencing. On the other hand, excessively low voltage can increase the likelihood of DNA adhering to the pore wall, impairing device performance and reliability. In 2012, Luan et al. implemented a more refined approach to control DNA translocation speed: feedback regulation of the applied electric field [89]. When DNA enters the pore, the open-pore current typically decreases due to physical volume exclusion, which reduces the number of ions passing through the cross-section per unit time. However, DNA also carries counterions along with it, and under low-ionic-strength conditions this can lead to a detectable increase in current as the molecule translocates. As illustrated in Figure 4a, once this signal is detected, a fast computational feedback loop can rapidly reduce or even reverse the bias voltage, thereby slowing down DNA passage. Moreover, by applying repeated, non-periodic reversals of the electric field, DNA can be made to oscillate back and forth within the nanopore, significantly improving the accuracy of detection.

Just as moving a stick can be controlled by either pulling from one end or pushing against the other, slowing DNA translocation can be achieved not only by reducing the driving force but also by increasing the resisting force [17,80,81]. Raising the viscosity of the solution is one effective means of increasing such resistance. Fologea et al. first systematically examined the influence of viscosity on translocation time in 2005 [92]. In their study, DNA molecules were translocated through glycerol solutions with viscosities ranging from 1 to 5.3 cP (measured using a Gilmont Instruments model GV-2100 falling-ball viscometer), as illustrated in Figure 4b. They observed that in a 1.5 M KCl-TE buffer under a bias voltage of 120 mV, the current blockade amplitude produced by 3 kbp DNA was inversely proportional to the solution viscosity, whereas the translocation duration increased proportionally with viscosity.

The duration of DNA translocation is influenced not only by solution viscosity but also by the type and concentration of ions present [80]. As shown in Figure 4c, representative current traces for DNA translocating through 1 M solutions of KCl, NaCl, and LiCl are displayed from left to right. Kowalczyk et al. observed that while the event amplitudes were similar, the translocation time increased markedly as the salt was changed from KCl to NaCl and then to LiCl. For 1 M solutions, the experimental translocation time ratios for dsDNA were KCl:NaCl:LiCl ≈ 1:1.7:4.8. They further noted that higher LiCl concentrations led to longer translocation times, with ratios of approximately 1:1.5:2 for 1 M, 2 M, and 4 M LiCl. This trend can be rationalized by the reduced electrophoretic capture probability of a less-charged object at a fixed voltage [81,93]. To explain these observations, the authors proposed a theoretical model in which the nanopore system is treated as a one-dimensional channel containing a series of periodic energy barriers. Each energy trough corresponds to a binding site on the DNA, with the periodicity and number of barriers (about 20 binding sites per 64 nm) reflecting the charge-distribution characteristics of real DNA. Higher barriers require more energy for an ion to detach from a binding site, indicating stronger ion–DNA binding. By systematically varying the barrier height, they examined how the ratio of the average force exerted by ions on the barriers to the maximum possible force from the external electric field changed. When the barrier is low, ions barely sense its presence; they rapidly cross the barrier under the electric field, spend little time at each site, and transfer almost no momentum to the DNA. In contrast, when the barrier is high, ions must acquire sufficient energy from the external field to escape the potential-energy trap. During this process, nearly all of the electric-field force is transferred via the ions to the barrier itself. The resulting counterion force largely opposes the electrophoretic force that drives DNA translocation, thereby reducing the net driving force on the DNA. Because lithium ions interact most strongly with DNA, they generate the largest counterion force, leading to the longest translocation times observed in LiCl electrolyte.

In 2022, Chau et al. proposed that a cooperative effect between salt and polyethylene glycol (PEG) components modulates signal enhancement and may influence the detection of translocation events [31], building upon earlier findings that viscosity can slow DNA translocation [80] and that LiCl also decelerates the process [80]. In their experiments, a series of alkali-metal halide solutions were prepared, each at 0.1 M concentration in 50% (*w*/*v*) PEG 35K. Linearized 4.8 kb DNA was diluted in 0.1 M KCl and translocated under a −500 mV bias. The results showed that electrolyte properties affect both the magnitude of current peaks and the residence time of single-molecule events (Figure 4d,e), which may arise from differences in cation-anion mobility. CsBr produced the strongest current-peak amplification but the shortest residence time among the tested salts, whereas LiCl gave the greatest increase in average residence time but the smallest increase in current amplitude. A general trend emerged: heavier salts such as CsBr, KI, and CsI strongly amplify current peaks, whereas lighter salts like LiCl, LiBr, and NaF typically exhibit longer residence times. The decrease in residence time and increase in current peak correlate with increasing anion atomic number (e.g., from KF to KCl to KBr and KI), likely due to mobility differences between cations and anions [94,95,96]. The authors also noted distinct effects of Li^+^ and Na^+^ compared to K^+^ and Cs^+^, as seen in population scatter plots (e.g., LiCl and NaCl vs. KCl and CsCl), suggesting that intrinsic salt properties underlie the variation in translocation characteristics. Earlier work by Papke et al. indicated that the electrolyte lattice energy, defined as the energy needed to separate one mole of an ionic solid into gaseous ions, can be used to estimate a salt’s propensity to interact with PEG. This energy is inversely related to molar mass [97]. Chau et al. found that the average current peak was inversely proportional to lattice energy, while the average residence time increased with lattice energy (Figure 4d,e). Overall, electrolyte properties are the main determinants of dsDNA translocation dwell time and current magnitude. These differences can be explained by salt-PEG interactions, approximated by lattice energy, indicating that synergistic electrolyte-PEG effects are key to the observed signal enhancement. Chau et al. attribute this coordination to the strong cation-chelating ability of PEG [98], and they further ascribe the signal variations among Li^+^, Na^+^, K^+^, and Cs^+^ solutions to differing degrees of interaction between these cations and the ether-oxygen groups of PEG [99].

Importantly, Chau also observed “current enhancement” or “conductive events” (CEs) during transport, where current exceeded baseline levels. Moreover, this phenomenon was not confined to specific polymer-electrolyte systems. The physical origin of such CEs has been systematically investigated. Lastra et al. challenged the conventional “molecule-centric” view that CEs arise solely from counterions carried by the analyte. By studying DNA and protein translocations in nanopipettes under various low-salt and asymmetric salt conditions, they proposed a “pore-centric” theory [101]. In this model, CEs result from an imbalance of ionic fluxes, leading to charge density polarization and a local change in the pore potential ($V_{\mathrm{pore}}$), rather than from the analyte itself. This mechanism explains why CEs are also observed for net-neutral proteins.

Further supporting the complex nature of translocation signals, Sulaiman et al. demonstrated significant differences in event characteristics for molecules moving “into” versus “out of” an asymmetric nanopore [102]. They also showed that signal enhancement analogous to that seen in PEG solutions can be induced by asymmetric salt conditions or by hydrogel-filled pores. These findings collectively indicate that the direction of translocation and the local ionic environment, particularly asymmetry, are critical parameters governing event polarity and magnitude.

Beyond electrical and chemical means, externally applied hydrostatic pressure provides an additional degree of freedom to control DNA translocation. A pressure bias across the membrane generates a pressure-driven flow (PDF) of the solvent. By carefully choosing the direction of this flow, one can create a counter-flow that opposes the electrophoretic force acting on the DNA [103,104]. This force balance can be described by:

$$F_{net}=F_{EP}-F_{PDF}=q_{DNA}E-\gamma \nu_{PDF}$$

where $q_{\mathrm{DNA}}$ is the effective charge of the DNA, E is the electric field, γ is a friction coefficient, and $v_{\mathrm{PDF}}$ is the velocity of the pressure-driven flow.

By tuning the pressure, the net force on the DNA can be reduced to near zero, dramatically slowing down the translocation process. More importantly, if the pressure is increased beyond a critical threshold where $F_{\mathrm{PDF}}>F_{\mathrm{EP}}$, the net force reverses direction. In this regime, DNA molecules that have been captured by the electric field can be pushed back out of the pore, effectively turning off translocation. This allows for a controllable on/off switch for DNA passage, which is invaluable for fundamental studies of capture dynamics and for developing new sensing modalities that require repeated interrogation of a single molecule.

This pressure-based technique is particularly attractive because it is compatible with standard microfluidic setups and does not require complex chemical modifications of the pore or the solution.

#### 3.2.2. Leveraging the Geometric Properties of Nanopore

As mentioned earlier, electron beam etching of h-BN nanopores preserves N-terminated edges, creating triangular pores on the h-BN film (Figure 4e) [50]. Unlike circular nanopores in other materials, the two-dimensional triangular nature of h-BN voids confers distinct translocation kinetics to DNA. Liu et al. observed a bimodal distribution of DNA translocation within h-BN pores. Molecular dynamics simulations confirmed that interactions between DNA and the triangular pore edges (van der Waals and electrostatic forces) are the primary cause of slow translocation (Figure 4f) [100,105]. Traditional models assume uniform ion mobility within and near the pore, yielding the formula [106,107]:

$$G=\sigma_{bulk}{\left[\frac{4L}{\pi d^{2}}+\frac{1}{d}\right]}^{-1}$$

where G is the nanopore conductivity, σ_bulk_ is the bulk solution conductivity, L is the effective membrane thickness, and d is the equivalent nanopore diameter. Liu et al. proposed a modified model [50]:

$$G=\sigma_{bulk}{\left[\frac{1}{\alpha_{pore}}\frac{4L}{\sqrt{3}a^{2}}+\frac{2\epsilon }{C_{A}}\right]}^{-1}$$

where a is the side length of the triangular pore, and C_A_ is the capacitance of the triangular conductive plate calculated using the moment method. The introduced parameter α_pore_ is a scaling factor representing the ratio of ion conductivity within the pore to the bulk conductivity. Experimental fitting yielded α_pore_ ≈ 0.3, indicating that ions undergo strong scattering during migration within triangular pores, resulting in an effective conductivity of only 30% that of the bulk phase. This may stem from the enhanced scattering effects of the pores’ sharp edges and corners on ions. Such intense ion scattering causes a significant reduction in the effective ionic conductivity within the pores. This is the fundamental reason for the slower DNA translocation rate and the smaller current blockage amplitude.

#### 3.2.3. Using Lasers to Alter the Surface Charge of Nanopore

In 2013, Fiori et al. developed a distinctive approach to control the translocation kinetics of biomolecules through nanopores. They focused a low-power (a few milliwatts) green laser (532 nm) directly onto the nanopore, which induced a reversible negative surface charge on the silicon nitride (SiN_x_) surface with a density reaching up to 1 C/m^2^. The modest thermal output of such low-power irradiation was insufficient to cause temperature changes that could affect DNA translocation [108,109]. Therefore, the observed increase in ionic current was attributed to a direct photoconductive interaction between the light and the silicon nitride membrane. This effect proved fully reversible on a submillisecond timescale. The enhanced surface charge, in turn, strengthened the electroosmotic flow within the pore. This flow was oriented opposite to the translocation direction of negatively charged DNA or proteins, thereby significantly slowing their passage through the nanopore [110,111]. Laser treatment is merely one method for altering the surface charge of nanopores. A more mainstream approach involves chemically modifying the nanopore surface, which will be discussed in Section 3.2.4.

#### 3.2.4. Modifications to the Nanopore via Chemical Functionalization

The fundamental concept behind slowing DNA translocation through nanopore modification relies on altering the physicochemical properties of the nanopore surface via chemical functionalization. This enhances interactions between DNA and the pore wall or modifies the hydrodynamic environment inside the pore, ultimately achieving controlled deceleration of DNA passage [112]. Depending on the underlying mechanism, modification strategies can be categorized into several types, including charge modification [113,114,115,116,117,118,119], hydrophobic modification [120,121,122,123,124,125,126,127], biomimetic modification [128,129,130,131], and other related approaches [54,132].

Currently, charge modification is the most widely employed approach. Its underlying mechanisms involve two primary effects: (1) the negatively charged DNA backbone experiences strong electrostatic attraction to positively charged pore walls, thereby increasing translocation resistance [118,119]; (2) intrinsically negatively charged nanopores generate an electroosmotic flow directed toward the cathode, which opposes the electrophoretic movement of negatively charged DNA. Enhancing this counter-directed electroosmotic flow or reducing/reversing any co-directed flow weakens the net electric field force acting on DNA, slowing its translocation speed [113,114,115,116,117].

**Figure 5:**
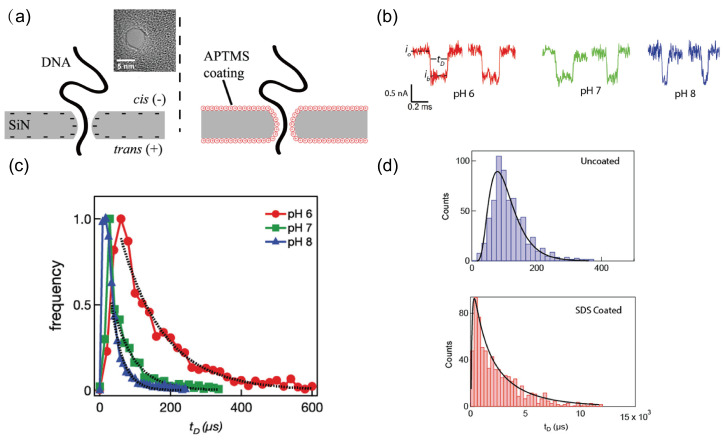
(**a**) After surface modification with APTMS, the nanopore surface becomes positively charged. (**b**) Typical transport events of 1 kbp DNA through APTMS-coated nanopores at pH values of 6.0, 7.0, and 8.0 show that residence time decreases as pH increases. (**c**) *t*_D_ histograms at pH 6.0, 7.0, and 8.0 with corresponding exponential time scales *t*_pH6_ = 118 μs, *t*_pH7_ = 46 μs, and *t*_pH8_ = 29 μs. Panels (**a**–**c**) are reproduced with permission from ref. [117]. © 2012 American Chemical Society. (**d**) Bar chart showing translocation time distributions for standard nanopores (top) and SDS-modified nanopores (bottom). After coating with sodium dodecyl sulfate, translocation speed slowed by a factor of 34. Reproduced from ref. [118]. CC-BY-NC-ND 4.0.

In 2012, Anderson et al. developed a method to precisely tune nanopore wall charge to modulate DNA translocation [117]. As illustrated in Figure 5a, they functionalized nanopores with 3-(aminopropyl)trimethoxysilane (APTMS). When the pH is lowered below the point of zero charge, the surface charge reverses, imparting a slight positive charge. By adjusting the pH, the surface charge of the nanopore could be conveniently and rapidly modified, thereby altering the interaction forces between the nanopore and DNA. Figure 5b,c show that decreasing the pH led to longer DNA translocation times.

In contrast to Anderson’s strategy of directly altering surface charge, Soni et al. leveraged changes in electro-osmotic flow (EOF) to influence DNA translocation [118]. They coated silicon nitride (SiN_x_) nanopores with the anionic surfactant sodium dodecyl sulfate (SDS), which formed a negatively charged monolayer on the pore surface, substantially increasing surface charge density. The adsorbed SDS attracted more sodium ions, thereby strengthening the EOF within the pore to a magnitude comparable to the applied electric field force. As shown in Figure 5d, the translocation rate slowed by a factor of 34 after SDS coating.

Some researchers have proposed slowing DNA translocation by modifying nanopore hydrophobicity. It should be noted that while DNA exhibits stronger interactions with hydrophobic surfaces, such interactions are generally undesirable because they can lead to nonspecific adsorption and eventual pore blockage [121,133,134]. Yin et al. first introduced amino groups onto nanopores using alkylsilane reagents, employed glutaraldehyde as a crosslinker, and finally covalently attached poly-L-lysine (PLL) [121]. PLL is a hydrophilic polymer whose modification reduces the contact angle and significantly enhances surface hydrophilicity. This helps keep the pore channel well-hydrated, ensuring reliable filling with electrolyte under varied conditions. In aqueous solution, the hydrophilic PLL chains become fully hydrated and extended, allowing the positively charged amino groups (-NH_3_^+^) along the polymer backbone to flexibly “grab” negatively charged DNA molecules as they pass. Due to current fabrication limitations, achieving sufficiently small diameters in solid-state nanopores remains challenging [135,136]. Researchers have therefore explored coating the interior of nanopores to effectively reduce their effective diameter. Wanunu et al. tested nanopores ranging from 5.0 nm to 2.7 nm and observed that DNA translocation through the 2.7 nm pore was nearly an order of magnitude slower than through the 5.0 nm pore [137]. This finding provides a theoretical basis for using interior coatings to narrow the pore and thereby decelerate DNA transit. Building on this, Akahori et al. reduced the nanopore diameter from 4.5 nm to 2.3 nm via atomic layer deposition (ALD), achieving a more than 16-fold decrease in the translocation rate of single-stranded DNA [138]. A drawback of this strategy, however, is that it tends to lower DNA capture efficiency, which is often detrimental in sequencing applications.

The diverse chemical modification strategies discussed above—whether altering charge, hydrophobicity, or effective pore size—ultimately function by tuning the fundamental chemical interactions between the DNA analyte and the nanopore surface. A systematic understanding of these interactions is essential for moving beyond empirical modifications toward a rational design of nanopore interfaces. These interactions can be broadly categorized into three main types:

1. Electrostatic Interactions: In aqueous electrolytes, most native oxide and nitride surfaces (e.g., SiN_x_, SiO_2_) acquire a surface charge, typically negative at physiological pH. The negatively charged DNA backbone therefore experiences a long-range repulsive force. As highlighted by the work of Anderson et al. and Soni et al. (Figure 5) [117,118], engineering the surface to introduce positive charges (e.g., via APTMS) can reverse this repulsion into attraction, increasing translocation resistance. Conversely, enhancing the native negative charge (e.g., with SDS) strengthens electroosmotic flow, which can oppose DNA’s electrophoretic motion. Electrostatic forces are thus a powerful and tunable knob for controlling capture and translocation dynamics.
2. Hydrophobic Interactions: This interaction is dominant for pristine, atomically flat surfaces like graphene. The hydrophobic effect drives the physisorption of DNA bases onto the surface to minimize their contact with water, leading to strong, yet non-specific, binding [38,139]. While this strong interaction can significantly slow DNA translocation, as observed in some graphene and BN nanopore experiments, it is also a primary cause of severe pore clogging. This trade-off underscores why hydrophobic surfaces often require passivation with hydrophilic coatings (e.g., PEG, pyrene-ethylene-glycol) to create a low-friction, antifouling interface, as discussed in Section 3.3.
3. Hydrogen Bonding: Surfaces bearing hydrogen bond donors or acceptors—such as the silanol groups on SiN_x_, the hydrophilic functional groups on modified BN, or polymer coatings like PLL—can form transient hydrogen bonds with the DNA bases and backbone. This interaction is generally weaker than hydrophobic adsorption but can contribute to a “stickiness” that slows translocation. In hydrogel-filled nanopores (HFNs), the extensive hydrogen-bonding network of the polymer matrix with water and ions creates a viscous, confined environment that dramatically increases friction and slows DNA motion, an effect distinct from direct DNA-pore wall interactions [140].

In practice, DNA translocation is governed by the complex interplay of these forces, which collectively define the free energy landscape experienced by the molecule as it traverses the pore. A deep understanding of this landscape is crucial for rationally designing nanopore surfaces. The goal is to achieve a delicate balance: harnessing interactions (e.g., electrostatic attraction or steric hindrance) to slow DNA for enhanced temporal resolution, while simultaneously minimizing non-specific, strong adsorption that leads to clogging and compromises the sensor’s reliability and throughput.

#### 3.2.5. Embedding Biological Nanopores into Solid-State Nanopores

Embedding biological nanopores such as MspA [141], phi29 [142], and α-hemolysin [128,131] into solid-state nanopores has emerged as an active research direction in recent years. Biological pores offer atomically precise structures and genetic programmability [143], whereas solid-state nanopores provide mechanical robustness, tunable pore dimensions, and ease of integration [144]. Each system, however, has distinct limitations: biological pores like α-hemolysin (αHL) rely on fragile lipid bilayers for support and are difficult to integrate, while fabricating solid-state nanopores with sub-nanometer precision remains challenging. These constraints can be addressed by inserting individual αHL pores into solid-state nanopores.

**Figure 6:**
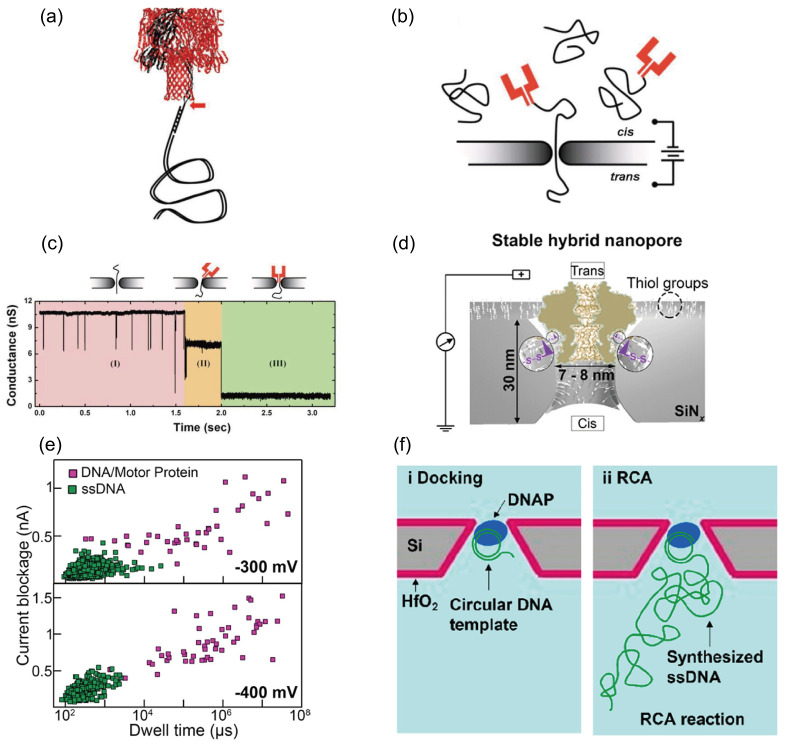
Embedding biological nanopores into solid-state nanopores (**a**) An αHL heteroheptamer with a 3 kbp dsDNA attached via a 12-nt oligomer to one protein subunit. The arrow indicates the position of the disulfide at the connection point. (**b**) Experimental setup, in which protein-conjugated dsDNA is electrophoretically translocated through a narrow solid-state nanopore. (**c**) A typical event wherein an αHL protein pore inserts into a SS-nanopore. The figure shows unconjugated dsDNA translocations (I), followed by a brief plateau indicating “pre-insertion” (II), and finally a stable, low conductance level (III). A voltage of V = −600 mV was applied to the cis chamber. Top: sketches of the three phases in the insertion process. Hybrid pore formation by directed insertion of alpha hemolysin into solid-state nanopores. Panels (**a**–**c**) are reproduced with permission from ref. [128]. © 2010 Springer Nature. (**d**) Insertion of the CD/N G20c portal protein into the thiolated SiN_x_ nanopore and formation of disulfide bridges between the protein and the pore wall ensures a highly stable hybrid nanopore. (**e**) Scatter plot of dwell time and current blockage for hybrid nanopores and solid-state nanopores. Hybrid nanopores slow DNA translocation speed by five orders of magnitude. Panels (**d**,**e**) are reproduced with permission from ref. [129]. © 2022 American Chemical Society. (**f**) Schematic illustration of docking of the phi29−template complex onto a nanopore and subsequent in situ RCA to synthesize ssDNA after docking. Reproduced from ref. [142]. CC-BY 4.0.

In 2010, Hall et al. engineered a single monomer of the αHL heptamer to include an additional 11-amino-acid loop at the β-barrel apex [128]. This loop contained a cysteine residue linked via a disulfide bond to a 12-base thiol-derivatized DNA oligonucleotide, which served as an attachment point for long dsDNA molecules with complementary single-stranded overhangs. The resulting polyionic tail guided the αHL pore into the solid-state nanopore with defined orientation (Figure 6a). As shown in Figure 6b, the solid state nanopore served as the sole conductive pathway between two fluid reservoirs, similar to conventional solid state devices. Applying a potential across the membrane generated a tightly confined electric field within the pore, which drove the transport of charged molecules. A key feature was the pore diameter, which ranged from 2.4 to 3.6 nm. This size was sufficient to allow the dsDNA guide and the stem of the mushroom-shaped αHL protein to enter, but remained too narrow for the larger cap region of αHL to pass. As a result, the dsDNA molecule translocated through the nanopore, pulling the attached αHL protein until it was mechanically arrested at the pore constriction.

In DNA translocation experiments, when a 100-base oligonucleotide (1 ng/μL) was added to the cis chamber, the recorded pulse signal is shown in Figure 6c. The conductance blockade (ΔG) for these events exhibited a bimodal distribution, consistent with earlier measurements of αHL in lipid bilayers [145]. Moreover, the peak of the residence-time distribution occurred at 360 μs, closely matching the previously reported characteristic dwell time of 330 μs [145]. This value is significantly longer than typical DNA translocation times observed in unmodified solid-state nanopores.

Building upon our previous work, in this study Mojtabavi et al. enhanced the performance of hybrid nanopores by “corking” the G20c portal protein into a SiN_x_ nanopore with “lipid-free hybrid” [129]. They employed a silane coupling agent that covalently bonds to SiOx-rich surfaces to form durable self-assembled monolayers (SAMs). The surface was thiol-functionalized by treating the SiN_x_ membrane with 2,2-dimethoxy-1-thio-2-silocyclopentane (silylsulfide). Driven by bond energy differences and ring strain release, the silylsulfide rapidly reacted with the surface via an open-ring click reaction [117,146,147,148]. Concurrently, they mutated a leucine residue in the native G20c protein to cysteine [149]. The chemically modified solid-state nanopores were assembled into a fluidic electro-mechanical measurement cell, where engineered G20c proteins were introduced into the buffer solution. Subsequently, a positive voltage was applied across the membrane. Due to the bipolar charge distribution on the external surface of the G20c gatekeeper protein, it was driven by electrophoresis and electroosmotic flow in a specific direction under the electric field, ultimately “plugging” into the solid-state nanopore of matching dimensions. After the protein was inserted into the pore, the catalyst Cu(phthalocyanine)_2_ was added. This catalyst promotes an oxidation reaction between the sulfhydryl groups on the protein surface and those on the pore walls, forming a robust disulfide bond (Figure 6d) [129,150]. With G20c’s assistance, DNA translocation slows by five orders of magnitude (Figure 6e), and because the device employs a lipid-free embedding method, it can withstand higher voltages than conventional lipid-embedding techniques. Li et al. first modified nanopores with HfO_2_ to reduce their diameter, enabling phi29 to snugly dock within the nanopores [142]. When phi29 docks onto a pore, it blocks Ca^2+^ ion flow, causing the corresponding pore’s fluorescence signal to weaken or disappear for up to several seconds. Once docking is stable, enzymatic activity is maintained. After voltage removal and substrate (dNTPs) provision, phi29 initiates activity, synthesizing DNA single strands in situ using circular DNA as a template. Since DNA displacement post-docking relies on enzymatic control rather than electrophoretic forces, DNA translocation speed is significantly reduced (Figure 6f).

#### 3.2.6. Precision Control via Spatial Tethering and External Forces

While modifying the pore or solution conditions can slow translocation, these methods often lack the precision needed to control the motion of an individual molecule deterministically. An alternative paradigm involves tethering the molecule of interest and using an external actuator to control its interaction with the nanopore.

**Figure 7:**
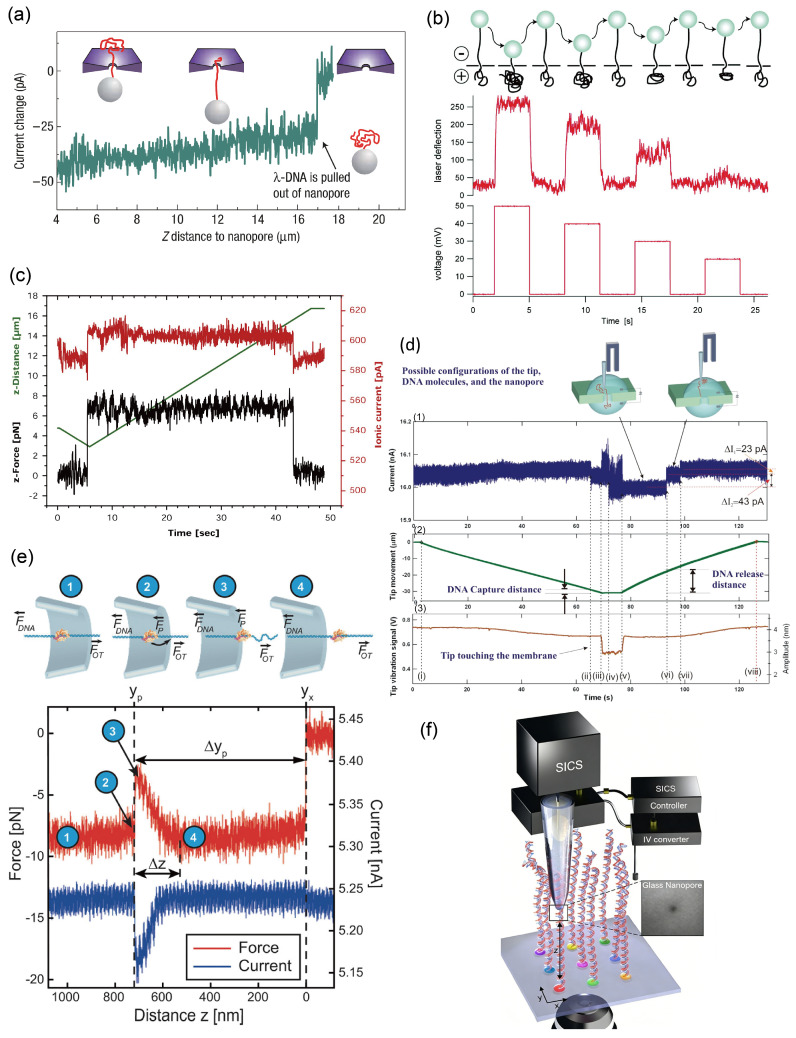
Controlling DNA translocation through external forces. (**a**) Schematic of DNA translocation through a nanopore controlled by optical tweezers, achieving a translocation speed of 30 nm/s under optical tweezers control. Reproduced from ref. [151]. © 2006 Nature Publishing Group. (**b**) DNA attached to a trapped bead is held near the nanopore. Once the DNA is threaded, it can be held in the pore at low applied voltage. Small increases in voltage cause small changes in displacement of the bead with respect to the trap center. This diagram illustrates DNA moving back and forth through the nanopore under the device’s action. Reproduced from ref. [152]. © 2007 American Chemical Society. (**c**) Dynamic translocation of ligand-complexed DNA through solid-state nanopores with optical tweezers. Reproduced from ref. [153]. © 2010 IOP Publishing Ltd. (**d**) Parameters measured in the SSN-TFFSP apparatus. (1) Current through the nanopore in 1 M KCl solution with 60 mV bias voltage. Current dropped when DNA molecules were captured by the nanopore, and current recovered to its original value when the DNA was released from the nanopore. (2) TFFSP tip’s movement. (3) Tuning fork’s voltage signal after 1000 times amplification as the tip approaches and lifts from the nanopore surface. The right axis is the vibration amplitude converted by 0.18 V/nm. Reproduced from ref. [154]. © 2013 American Chemical Society. (**e**) Typical force and current curves for RNAP. Reproduced from ref. [155]. © 2016 American Chemical Society. (**f**) Schematic of Controlled Single-Molecule Transposition Using Nanopore-Based Scanning Ion Conductance Spectroscopy (SICS). Reproduced from ref. [156]. © 2023 Springer Nature.

In 2006, Keyser et al. first applied optical tweezers to nanopore sequencing [151]. As illustrated in Figure 7a, optical tweezers can reduce DNA translocation speed by up to five orders of magnitude and even bring the molecule to a complete halt. Building on this, Treagnir et al. later demonstrated that optical tweezers could not only regulate translocation speed but also enable repeated back-and-forth motion of DNA within the nanopore (Figure 7b) [152]. However, incorporating optical tweezers increases system complexity and introduces low-frequency thermal noise, which can compromise nanopore performance.

In 2010, Sischka et al. combined quantitative three-dimensional optical tweezers (OT) with electrophysiology to study the threading and controlled translocation of single λ-DNA molecules through a solid-state nanopore with millisecond temporal resolution [153]. Using this setup, they quantitatively examined the binding of RecA and individual peroxidase proteins to λ-DNA during dynamic translocation, measuring both the effective forces and the corresponding ionic currents as DNA was pulled inward and outward through the pore (Figure 7c).

Hyun et al. developed a system called SSN-TFFSP, whose key innovation is the integration of a solid-state nanopore with a tuning-fork-based force-sensing probe (Figure 7d) [154]. One end of λ-DNA was anchored via a biotin-streptavidin bond to a metal-coated probe tip mounted on a quartz tuning fork, which acts as a highly sensitive force sensor capable of sub-nanometer positional control in solution. The DNA-tethered probe was positioned near a nanopore under applied voltage. The external electric field then captured the free end of the DNA and pulled it into the pore. After the DNA was captured and stretched, the operator could slowly withdraw the probe from the nanopore using a nanopositioning system. This reverse motion resembles carefully threading a needle in reverse. The extraction speed is entirely user defined, enabling precise control over the DNA translocation velocity.

Bulushev et al. captured and manipulated polystyrene microspheres carrying DNA-protein complexes using optical tweezers. By applying voltage, they introduced DNA into nanocapillaries while simultaneously recording changes in current and force [155]. As shown in Figure 7e, the process unfolds in four stages. Before the protein enters the jump region, the force acting on it is comparable to that on the DNA. At the moment the jump initiates, the force exerted on the protein surpasses that on the DNA. Once the jump is complete, the degree of DNA stretching decreases. Following the jump, the DNA stretching returns to its pre-jump level. In summary, as the complex passes through the constriction, it produces a distinct jump signal, characterized by a force peak alongside a drop in current. This enables the controlled transport and characterization of various DNA-protein complexes.

With technological advancements, Leitao et al. developed a method of controlling translocations of single molecules with nanopore-based scanning ion conductance spectroscopy (SICS) [156]. DNA molecules were deterministically tethered to a glass surface via one end. By controlling the distance between the nanopore and the surface, the tip of the nanopore could be positioned to actively select a specific region of interest on a single DNA molecule. The molecule was then scanned back and forth through the pore by moving the stage (Figure 7f). This approach offers unprecedented control: the translocation velocity can be arbitrarily set (e.g., as low as 20 nm/s), and the same molecular section can be read thousands of times. By averaging these multiple reads, the signal-to-noise ratio was improved by two orders of magnitude compared to free translocations, enabling the detection of single-nucleotide gaps. This technique elegantly bypasses the stochastic nature of free translocations, transforming nanopore sensing from a passive counting tool into an active scanning probe.

Although many external-control methods have been proposed, their practical adoption has been limited by challenges such as high equipment complexity, substantial cost, thermal noise from optical tweezers, and the need for specialized expertise. Nevertheless, these approaches continue to provide researchers with unique avenues for investigating controlled molecular transport.

### 3.3. Clogging of the Nanopore

**Figure 8:**
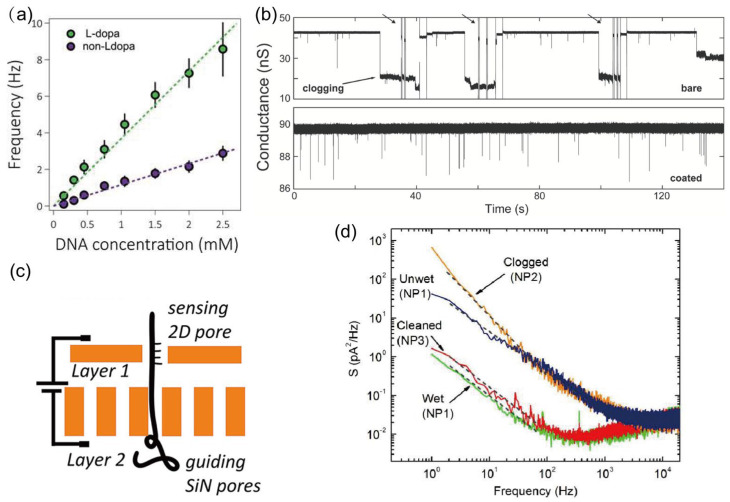
Reduction in DNA clogging. (**a**) Frequency of DNA translocation events in nanopores modified with levodopa (green) versus unmodified nanopores (purple). DNA interaction with nanopores is weakened after levodopa modification. Reproduced from ref. [157]. CC-BY-NC. (**b**) Comparison of ssDNA translocation between bare (top) and PEG (bottom) coated nanopore at pH 11.5 under voltage of 100 mV. The coated nanopore presents stable and smooth translocation. Reproduced from ref. [134]. © 2014 WILEY-VCH. (**c**) This figure illustrates a conceptual nanopore array scaffold design composed of large-aperture silicon nitride (SiN) pores, optionally supported by pillars, to direct DNA within the nanopore device into a constrained geometry. Reproduced from ref. [158]. © 2021 American Chemical Society. (**d**) The orange curve represents the typical PSD of pores irreversibly blocked during DNA translocation experiments, and even after two separate 30 min washes in piranha solution at 75 °C, it failed to regenerate a low-noise current trace. The red curve shows the PSD of a 11 nm (G = 47.0 ns) blocked pore cleaned using a 10 V, 200 ms pulse. Reproduced from ref. [159]. © 2012 IOP Publishing Ltd.

In practical nanopore applications, the persistent clogging of pores by adsorbed or trapped molecules remains a significant challenge. Several strategies have been developed to mitigate this issue, including surface passivation techniques to reduce nonspecific interactions. The surface properties of solid-state nanopores often promote nonspecific DNA-wall interactions, leading to pore blockages. Cai et al. functionalized solid-state nanopores with levodopa, which weakens DNA-pore adhesion [157]. Compared to unmodified pores, the linear-fit slope increased threefold, indicating that functionalization facilitates DNA entry into the nanopores (Figure 8a). Tang et al. reported that PEG-coated SiN nanopore devices reduce DNA clogging caused by surface adsorption (Figure 8b) [134]. Polyethylene glycol (PEG), a widely used polymer, self-assembles on SiN surfaces and suppresses nonspecific binding of DNA to the pore wall. Graphene nanopores, due to their extreme hydrophobicity, are particularly prone to severe nonspecific adsorption and clogging. Schneider et al. addressed this by non-covalently modifying graphene nanopore surfaces with pyrene-ethylene-glycol via self-assembly [38]. The pyrene moiety anchors the coating to the graphene, while the ethylene-glycol chains provide a hydrated, antifouling interface. Even single-stranded DNA, which tends to adhere more strongly than double-stranded DNA, can be detected through such modified graphene nanopores without significant clogging.

Another approach involves constraining the range of DNA motion through device-level design. Niedzwiecki et al. confined DNA within defined geometries using silicon-nitride pillars and surrounding nanofluidic structures (Figure 8c) [158]. Atomically thin two-dimensional pores provide optimal sensing sensitivity, while the integrated silicon-based pillars, trenches, and pore arrays collectively guide DNA translocation, limiting entropic wandering and thereby minimizing the likelihood of clogging.

A third strategy utilizes the application of short high-field pulses for in situ cleaning. Beamish et al. demonstrated that short, high-field electrical pulses can clean nanopores easily, rapidly, and in situ, avoiding risks of membrane damage and bypassing lengthy chip-handling procedures [159]. As shown in Figure 8d, the orange curve represents the power-spectral-density (PSD) trace of a pore that became irreversibly clogged during DNA translocation. Even after two separate 30 min washes in 75 °C piranha solution, a low-noise current trace could not be recovered. In contrast, the red curve shows the PSD of an 11 nm pore (conductance = 47.0 nS) that was blocked and then cleaned using 10 V, 200 ms pulses. In this case, just two pulses were sufficient to remove the noise source, restoring a clean nanopore surface with highly stable conductivity.

### 3.4. Noise

In practical nanopore applications, a core and nearly fundamental challenge faced by researchers is noise. Within the context of solid-state nanopore sequencing, “noise” is not a single adversary but a multifaceted phenomenon arising from a combination of intrinsic physical limits, material imperfections, and the inherently stochastic dynamics of the analyte itself. It is precisely this complex, multi-source noise that severely degrades the signal-to-noise ratio of solid-state nanopores, obstructing their ultimate aim of achieving single-base or single-amino-acid resolution. This reality compels us to confront several unresolved core questions in the field. As highlighted in the previous section on temporal resolution, the demand for higher measurement bandwidth to resolve fast DNA translocations directly amplifies the impact of all noise sources. The signal-to-noise ratio (SNR) becomes the central battlefield where the gains in speed are constantly challenged by increases in noise.

Importantly, the strategies for improving temporal resolution discussed in Section 3.2.1—particularly the manipulation of ionic strength and electrolyte species—are not independent of noise performance. They directly influence both thermal and 1/f noise, creating critical trade-offs that must be navigated for optimal device operation.

Lowering salt concentration, while effective at slowing DNA via enhanced electroosmotic flow [73,80], increases the pore resistance R. As thermal noise scales as $I_{rms}^{2}\propto 1∕R$, this directly elevates the baseline noise floor, potentially negating the benefits of slower translocation. Conversely, high-salt conditions reduce thermal noise but accelerate DNA, demanding higher measurement bandwidth and introducing other noise penalties.

The choice of electrolyte species (e.g., Li^+^ vs. K^+^) also modulates 1/f noise. Ions with stronger DNA binding affinity (Li^+^) exhibit longer translocation times due to stronger momentum transfer [80], but this same strong interaction leads to more frequent and energetic ion capture–release events at the pore surface—a primary mechanism for charge fluctuations and thus 1/f noise generation [160,161]. Furthermore, salt gradients designed to create electrokinetic traps can induce concentration polarization near the pore [74], potentially exacerbating charge fluctuations and elevating low-frequency noise.

Thus, the optimization of ionic conditions for temporal resolution cannot be performed in isolation; it must be co-optimized with noise performance to ensure that gains in translocation time translate to measurable improvements in signal quality.

#### 3.4.1. Thermal Noise

Thermal noise in solid-state nanopores, also known as Johnson-Nyquist noise or white noise, stems from the random thermal motion of charged ions within the nanopore channel and the surrounding electrolyte. This motion, also called Brownian motion, involves ions such as K^+^ and Cl^−^. In simple terms, even in the absence of DNA translocation, the random movement of these ions under an applied bias generates minute, rapid fluctuations in the ionic current flowing through the pore. This constitutes thermal noise. Its power spectral density is essentially uniform over a broad frequency range (hence “white” noise), indicating that the noise amplitude is similar across frequencies. At any instant, the noise current follows a Gaussian (normal) distribution. Because thermal noise arises in any resistive element whose temperature exceeds absolute zero (–273.15 °C), it is governed by fundamental physical laws and is therefore unavoidable in practice. The intensity of thermal noise can be quantified by a classical formula:

$$i^{2}=\frac{4k_{B}TB}{R}$$

where $i^{2}$ is the root-mean-square value of the noise current; $k_{B}$ is the Boltzmann constant (1.38 × 10^−23^ J/K); T  is the absolute temperature; B is the bandwidth of the measurement system; and R is the resistance of the nanopore [162,163]. This relationship indicates that thermal noise scales proportionally with temperature and measurement bandwidth, while being inversely proportional to the nanopore resistance. Lowering the operating temperature offers one route to reduce thermal noise, yet its practical impact is often limited, and it becomes unfeasible in devices that incorporate active proteins or require physiological conditions. Another option is to narrow the measurement bandwidth, but doing so restricts the detectable frequency range and can compromise the capture of detailed signal information, effectively trading temporal resolution for a quieter baseline. Since nanopore resistance is inversely proportional to pore diameter and directly proportional to membrane thickness, reducing the pore diameter could theoretically suppress thermal noise. However, fabrication techniques are approaching the physical lower limit of pore size (around 1 nm), and the pore must still accommodate the dimensions of the translocating molecule. Conversely, increasing membrane thickness would raise resistance and thus reduce noise, but this conflicts directly with the need for ultrathin membranes to achieve high spatial resolution in single-molecule sensing.

#### 3.4.2. 1/f Noise

The 1/*f* noise of solid-state nanopores, also termed flicker noise, refers to current or voltage fluctuations whose power spectral density is inversely proportional to frequency. As the name indicates, its noise power declines as frequency increases. Unlike thermal noise, which stems from the random thermal motion of ions, the origin of 1/*f* noise is more complex and is primarily linked to surface properties and dynamic interactions within the pore. Although its precise mechanism is not yet fully unified, the dominant explanation is charge-fluctuation theory. This theory posits that numerous energy “traps” or defects exist on the surface of solid-state nanopores (e.g., silicon nitride or silicon dioxide). Ions in solution (such as K^+^ and Cl^−^) are randomly captured by these surface states and later released after a characteristic dwell time. Each capture-and-release event slightly modifies the local charge environment on the pore wall, thereby modulating the overall conductance of the channel and producing a minute current step. The superposition of countless such random events collectively gives rise to the observed 1/*f* noise [160,161].

When DNA molecules translocate slowly through a nanopore, the resulting blockade signal resides in the low-frequency range. Strong 1/f noise can completely overwhelm this signal, making base recognition impossible. This interference represents the most critical impact of flicker noise. Additionally, 1/f noise manifests as slow baseline fluctuations and drift in current traces, which makes accurately measuring the amplitude and duration of current blockades extremely difficult. Since 1/f noise originates from surface interactions, mitigation strategies focus largely on surface engineering. Coating the nanopore interior with a thin, high-quality dielectric layer is the most effective approach. Using techniques such as atomic layer deposition (ALD), an ultrathin, amorphous material, for example ZnO, Al_2_O_3_, or TiO_2_, can be deposited onto the pore walls. This coating covers the original rough surface, eliminates most charge traps, and fundamentally reduces the frequency of ion capture and release events.

**Figure 9:**
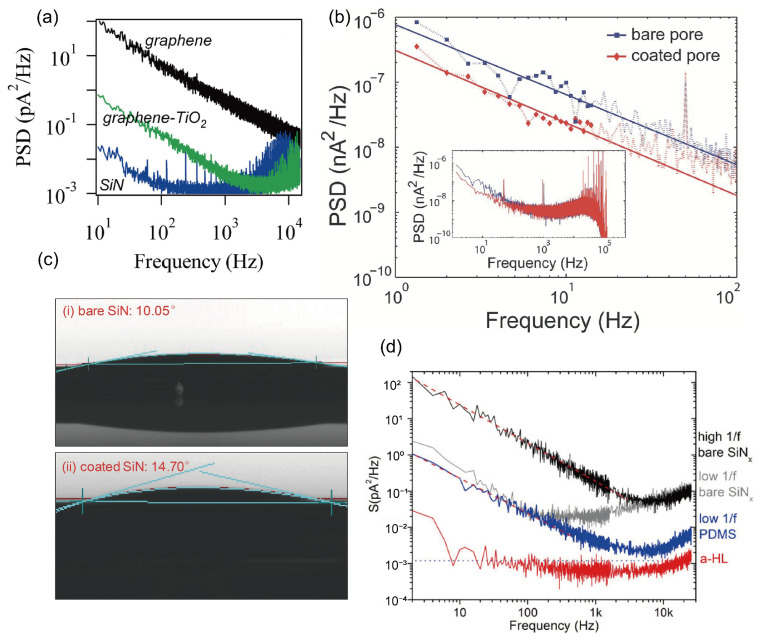
(**a**) Compared to exposed graphene nanopores (black), TiO_2_-coated graphene nanopores (green) exhibit a tenfold reduction in noise levels. Reproduced from ref. [59]. © 2010 American Chemical Society. (**b**) Comparison of power spectral density (PSD) between exposed (blue) and polyethylene glycol-coated (red) SiN nanopores. (**c**) Water contact angles of exposed (i) and polyethylene glycol-coated (ii) SiN nanopores. Panels (**b**,**c**) are reproduced with permission from ref. [134]. © 2014 WILEY-VCH. (**d**) Significant reduction in 1/f noise for conventional SiN nanopores treated with piranha solution (gray) compared to untreated conventional SiN nanopores (black). Reproduced from ref. [169]. © 2007 IOP Publishing Ltd.

Merchant et al. employed ALD to coat both sides of nanopores with TiO_2_ [59], which exhibits excellent hydrophilicity and deposits uniformly on graphene surfaces. This treatment was shown to reduce overall nanopore noise, particularly in the low-frequency range [164]. As illustrated in Figure 9a, TiO_2_-coated membranes exhibited about an order-of-magnitude lower noise compared to conventional graphene films. Similarly, Chen et al. demonstrated that ALD-deposited Al_2_O_3_ on nanoporous membranes induces surface passivation and reduces 1/*f* noise [165]. Park et al. likewise used ALD to deposit ZnO on nanopore membranes, achieving lower 1/*f* noise levels [166].

Tang et al. modified nanopores with PEG200, where PEG molecules bind to the SiN surface via hydrogen bonds and effectively shield the abundant silanol groups present on SiN [134]. The protonation and deprotonation of these groups vary with pH, leading to continuous fluctuations in surface charge, which are a primary source of 1/f noise [167]. This passivation dampens the pH-dependent response of the surface charge, thereby stabilizing it and reducing noise caused by charge fluctuations (Figure 9b). Moreover, the PEG coating increases the surface contact angle (Figure 9c) [134]. This helps minimize the unstable adsorption and desorption of water molecules and ions on the surface, processes that also contribute to 1/f noise [167]. Importantly, unlike some earlier coating methods [164,168], PEG200 modification does not cause significant reduction or drift in pore geometry or conductivity.

When nanopore surfaces are hydrophobic or contain surface defects, nanobubbles can form along the pore walls. These nanobubbles induce pronounced fluctuations and sudden steps in electrical conductance, generating substantial 1/*f* noise [164]. Consequently, Tabard-Cossa et al. proposed treating nanopores with piranha solution to remove nanobubbles and thereby suppress 1/*f* noise [169]. As shown in Figure 9d, untreated standard SiN nanopores exhibited significantly higher 1/*f* noise; after piranha-solution treatment, the noise level dropped markedly, matching the nearly identical 1/*f* characteristics observed below 100 Hz for polydimethylsiloxane (PDMS)-treated pores. Consistent with earlier findings, applying short high-field electrical pulses can also mitigate low-frequency noise associated with transient pore blockages [159].

#### 3.4.3. Dielectric Noise

**Figure 10:**
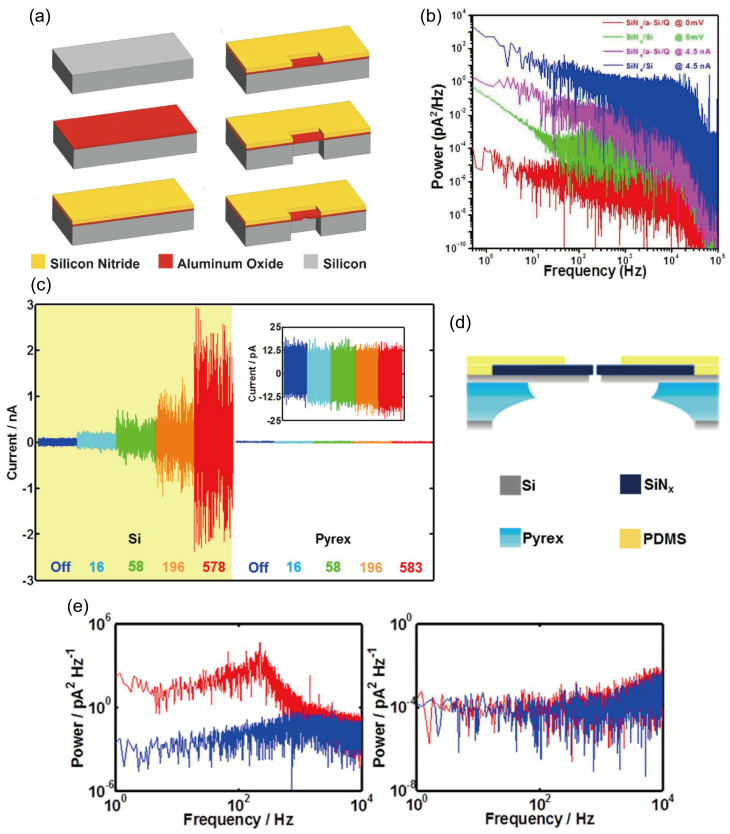
To reduce dielectric noise, (**a**) Al_2_O_3_ was deposited on silicon via ALD, followed by plasma-enhanced chemical vapor deposition (PECVD) of 500 nm low-stress silicon nitride (SiN) as a passivation layer. Reproduced from ref. [171]. © 2009 WILEY-VCH. (**b**) Power spectrum of SiN_x_ 20 nm on Si and a-Si 200 nm/quartz 200 μm measured in 1 M KCl while maintaining a pore current of 0 and 4.5 nA, respectively. Reproduced from ref. [172]. CC-BY 4.0. (**c**) Baseline ionic current at 0 mV, under laser illumination, for a ∼27 nm diameter nanopore in a Si-SiN_x_ (yellow background) and Py-SiN_x_ platform. Different color traces correspond to different laser powers, as indicated by the number (in μW units) beneath each trace. The inset is an expanded view of data for the Py-SiN_x_ device. (**d**) Schematic diagram of the PYREX substrate nanopore platform. (**e**) Power spectral density at 0 mV for the Si-SiN_x_ platform (left) and Py-SiN_x_ platform (right) with laser off (blue) and laser power at 578 μW (red). Panels (**c**–**e**) are reproduced with permission from ref. [173]. © 2015 American Chemical Society.

An ideal insulating (dielectric) material stores energy as charge in an electric field without dissipating power. Real-world materials such as silicon nitride (SiN_x_), however, are not perfect; they contain internal defects, dipoles, and other structural imperfections. When the electric field direction varies, the reorientation of molecular dipoles or the rearrangement of trapped charges lags behind the field change. This occurs even under a nominally constant bias, since ionic current fluctuations inherently contain a broad spectrum of frequency components. The resulting delayed response creates internal friction within the crystal lattice, converting a portion of the electrical energy into heat, a phenomenon known as dielectric loss. Consequently, a more accurate electrical model for a nanopore-membrane system is not a pure capacitor (C), but rather a parallel combination of a capacitor (C) and a resistor (R), which together account for both charge storage and energy dissipation. The dielectric noise can be described as:

$$S_{D}\left(f\right)=4kT\cdot Y_{f}=8\pi kTC_{n}D\cdot f$$

where D is the dielectric loss constant [170]. It is evident that materials with higher loss exhibit greater dielectric noise. As shown by the above formula, the magnitude of dielectric noise depends on the capacitance (C) and the dielectric loss constant (D), and it is directly proportional to both capacitance (C) and the dielectric loss constant (D). One method to reduce dielectric noise is to decrease capacitance. This can be achieved by coating the nanopore chip with insulating material or by minimizing the film size during fabrication, retaining only the minimum area necessary to support the nanopores. Venkatesan et al. deposited Al_2_O_3_ on silicon via ALD, followed by a 500 nm low-stress silicon nitride (SiN) layer via plasma-enhanced chemical vapor deposition (PECVD) as a passivation layer (Figure 10a) [171]. This helped reduce device capacitance and electrical noise, lowering noise levels by an order of magnitude. They attributed this to the reduced device capacitance (20 ± 5 pF), significantly lower than the capacitance of conventional SiN nanotunnels (300 pF). They further proposed that noise performance could be enhanced by combining device optimization with fluid isolation techniques using PDMS.

Another approach employs low-loss materials. Quartz is an ideal candidate due to its low loss constant. Leveraging the excellent insulating properties of quartz, Lee et al. developed a novel solid-state nanopore on a quartz substrate [172]. This structure consists of micrometer-sized holes fabricated on quartz and a freestanding silicon nitride (SiN) film only a few nanometers thick. Quartz exhibits a dielectric constant of 3.8 and a dielectric loss factor of 10^−4^, both significantly lower than the corresponding values for silicon (11.8 and 5–15 × 10^−3^, respectively). Figure 10b presents the power spectral density (PSD) curves of silicon- and quartz-based nanopores, measured with and without an applied voltage. The results clearly show that the quartz-based device achieves a reduced noise spectrum across the entire frequency range. Notably, at low frequencies, the quartz substrate substantially lowers the noise level regardless of the applied bias.

Combining the two strategies described earlier, namely applying an insulating coating and using a substrate with low dielectric loss, maximizes the reduction in dielectric noise. As illustrated in Figure 10c, Pitchford et al. developed a compliant platform consisting of nanopores on a silicon nitride (Py-SiN_x_) membrane supported by a glass substrate [173]. Owing to the high resistivity of Pyrex (400 MΩ·m), the Py-SiN_x_ platform exhibits low capacitance (5–10 pF in 1 M KCl buffer solution). Consequently, it achieves lower dielectric noise and input capacitance noise compared to Si-SiN_x_ platforms (fabricated on boron-doped silicon with a substrate resistivity of 1–30 Ω·cm). Figure 10d shows the baseline current measured at different laser powers. In comparison with Si-SiN_x_, the Py-SiN_x_ platform demonstrates visibly more stable baseline performance. Figure 10e presents the power spectral density (PSD) for both platforms with the laser off and at 580 μW laser power. Even under 580 μW illumination, the Py-SiN_x_ platform still maintains lower noise levels than the Si-SiN_x_ counterpart.

#### 3.4.4. Amplifier Noise

**Figure 11:**
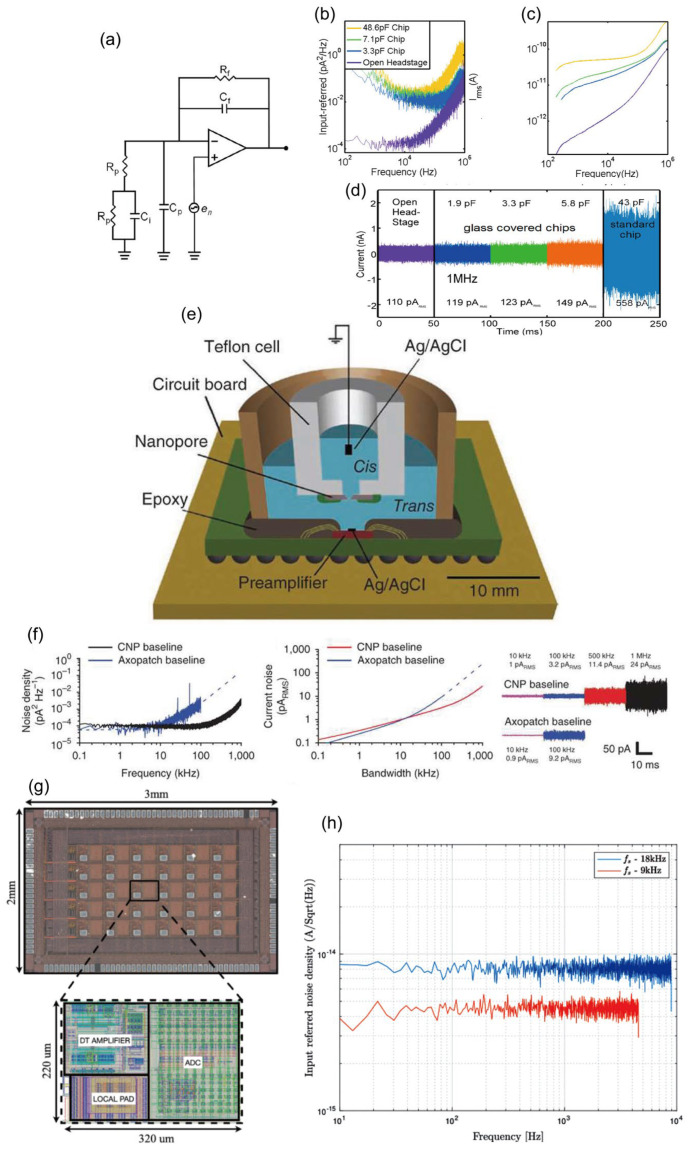
Reduce amplifier noise. (**a**) Equivalent circuit model for amplifier noise calculation. Reproduced from ref. [170]. CC-BY. (**b**) Input-referred noise power spectral density (PSD) for two glass-passivated nanopore chips with membrane capacitances of 3.3 and 7.1 pF. The PSDs for an unpassivated chip (with Cchip = 48.6 pF) and the open-headstage amplifier are also shown. (**c**) Irms calculated from the data in part a, as a function of signal bandwidth showing increase in noise proportional to capacitance at frequencies above 105 Hz. (**d**) Measured ion current temporal traces for several glass chips with capacitances Cchip = 0.69, 0.73, 1.1 pF, and 1.65 pF showing an amplifier-limited noise. A current trace from a 12 pF is shown for comparison. Panels (**b**–**d**) are reproduced with permission from ref. [176]. © 2014 American Chemical Society. (**e**) Schematic of the CMOS-integrated nanopore platform (CNP). (**f**) Corresponding reference current noise traces for the two amplifiers. Panels (**e**,**f**) are reproduced with permission from ref. [174]. © 2012 Springer Nature. (**g**) Microscope image of the array chip and detailed channel layout. (**h**) Open-input noise spectrum of the system at 9 kHz and 18 kHz sampling frequency. Panels (**g**,**h**) are reproduced with permission from ref. [177]. CC BY-NC-ND.

Amplifier noise is not a single type of noise, but rather arises from the combined effects of several physical mechanisms, primarily including: 1. Random thermal motion of electrons in all resistive components, also known as Johnson-Nyquist noise. 2. Random capture and release of charge carriers in semiconductor devices (e.g., the amplifier’s input transistor). 3. Discrete, random passage of charge carriers (e.g., ions) through nanoscale barrier potential wells. In the high-frequency range, the noise power spectral density (PSD) is proportional to f^2^, corresponding to amplifier noise [174]. Amplifier noise can be expressed as:

$$S_{A}\left(f\right)={\left(2\pi C_{t}e_{n}\right)}^{2}\cdot f^{2}$$

in which e_n_ is the voltage thermal noise, C_t_ is the total capacitance that consists of the nanopore capacitance (C_i_), feedback capacitance (C_f_), and other parasitic capacitance (C_p_), as shown in Figure 11a [170,175].

The above formula suggests two principal strategies for reducing amplifier noise: minimizing the total capacitance (Ct), and selecting or designing amplifiers with lower voltage thermal noise (En). Balan et al. implemented the first strategy by bonding a 100–200 μm thick glass layer onto a nanoscale chip surface and subsequently coating it with a thick silicone rubber layer [176]. Acting as an insulator, the glass layer substantially reduced the chip’s effective exposed area to the electrolyte, thereby lowering its capacitance from the conventional ~50 pF to the range of 1–5 pF. Figure 11b shows the ion-current noise input power spectral density (PSD) as a function of bandwidth for chips with capacitances of 3.3 pF and 7.1 pF, together with the noise spectrum of an uncoated glass chip (48.6 pF); for reference, the open-circuit preamplifier noise spectrum is also included. Figure 11c presents the integrated ion-current noise (I_rms) corresponding to the bandwidth function shown in Figure 11b, clearly demonstrating that noise decreases with capacitance.

In the following year, the same team reported a chip design and fabrication approach based on suspended silicon nitride membranes spanning holes in a glass substrate [178]. They introduced two innovative designs: a baseline chip achieving capacitance below 2 pF, and a refined two-step fabrication process that further reduced capacitance below 1 pF. Recognizing that the silicon substrate dominates the overall device capacitance, their strategy replaced the conventional silicon substrate with a hollow glass substrate. This modification lowered the total chip capacitance to below 1 pF, leading to a marked improvement in the noise performance of ion-current signals. Figure 11d displays ion-current time traces obtained from glass chips with capacitances of 0.69, 0.73, 1.1, and 1.65 pF, along with a reference trace from a 12 pF chip, directly demonstrating the superior noise-reduction capability resulting from their exceptionally low capacitance.

Rosenstein et al. moved away from commercial, standalone external amplifiers (e.g., the Axopatch 200B) and instead designed a CMOS-integrated nanopore platform (CNP), as shown in Figure 11e [174]. This integration reduced the distance between the nanopore and the amplifier from centimeter/millimeter scales to the micrometer scale, thereby minimizing wiring capacitance (Cw) to near-negligible levels. In addition, they coated most of the amplifier surface with a thick epoxy-based photoresist layer, achieving Cw < 1 pF. By implementing a servo loop with an active low-noise current source to replace the conventional feedback resistor, they attained higher gain performance and lower noise. Figure 11f compares the baseline noise spectrum of the CNP system with that of a similarly configured open-top Axopatch 200B. At measurement bandwidths below 10 kHz, the Axopatch exhibits lower noise than the CNP, a difference attributed to white noise originating from the on-chip components of the CNP. Above 10 kHz, however, the CNP shows lower noise, and this advantage becomes more pronounced with increasing bandwidth.

Separately, Dawji et al. proposed a signal conditioning array for nanopore-based DNA sequencing [177]. The array consists of 30 channels, each containing a discrete-time (DT) amplifier and a successive-approximation-register analog-to-digital converter (SAR ADC) within the same pixel (Figure 11g). The DT amplifier operates at a transimpedance gain on the order of gigaohms (GΩ), enabling the detection of picoampere-level currents. This chip forms a complete, integrated system that incorporates both electrodes and readout circuitry, establishing a foundation for high-throughput DNA sequencing experiments. As shown in Figure 11h, the system demonstrates low open-circuit input noise at both 9 kHz and 18 kHz sampling frequencies.

Enhancing the sensitivity and specificity of solid-state nanopore detection requires the pore to sensitively and accurately identify specific target molecules, such as particular proteins or DNA sequences, while effectively filtering out background molecules. This capability is critically important for practical applications, as real-world samples are often complex mixtures. Surface chemical modification and functionalization represent one of the most direct and versatile strategies, focusing on chemically grafting recognition elements onto nanopore surfaces. By utilizing the surface chemistry (e.g., hydroxyl or amino groups) of solid-state materials like SiN or SiO_2_, specific ligands can be covalently anchored to the pore entrance and inner walls through silanization or other coupling reactions [115,168,179,180,181].

**Figure 12:**
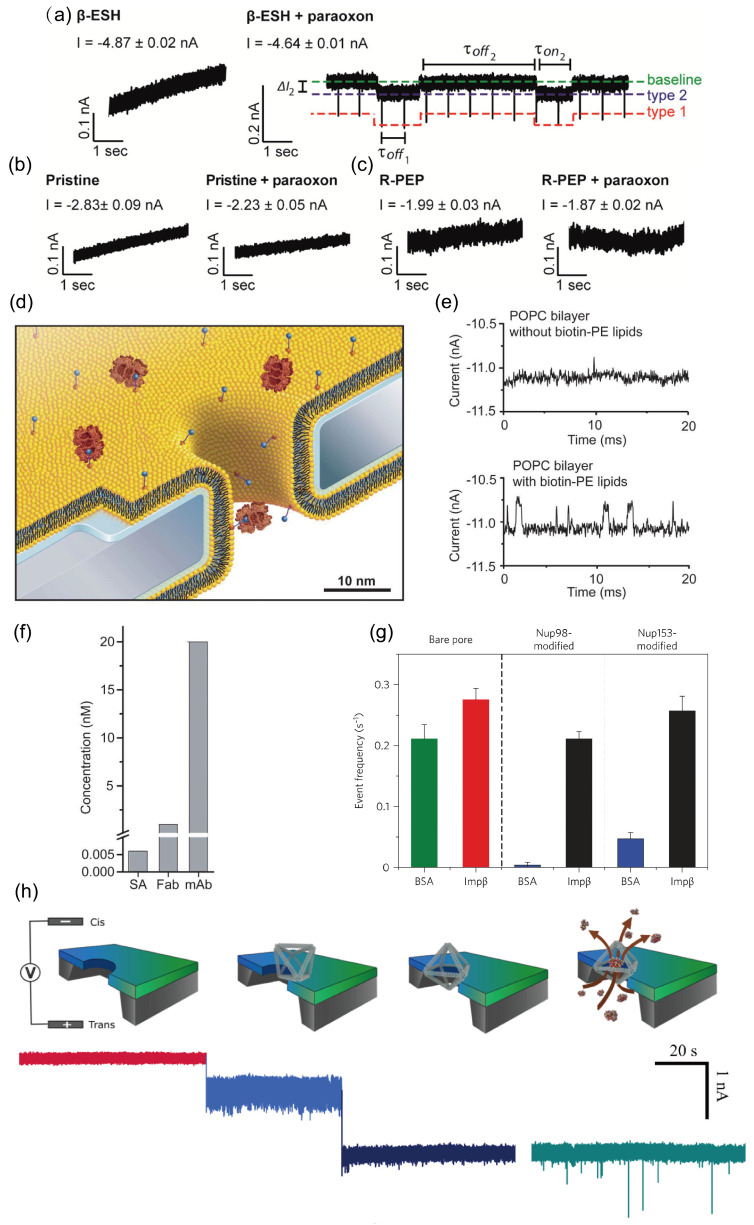
Enhancing the sensitivity and specificity of nanopores (**a**) Current events from a nanopore modified with the peptide (Glu-Ser-His) without paraoxyphosphonium (left) and with paraoxyphosphonium added (right). (**b**) Current events from an unmodified nanopore without paraoxyphosphonium (left) and with paraoxyphosphonium added (right). (**c**) Current events in nanopores modified with random peptide (R-PEP) without p-oxyphosphine (left) and with p-oxyphosphine added (right). Panels (**a**–**c**) are reproduced with permission from ref. [179]. © 2014 American Chemical Society. (**d**) Cartoon, drawn to scale, illustrating binding of streptavidin (large red) to specific lipid-anchored biotin-PE (blue circles) followed by single molecule translocation of the anchoredcomplex through the nanopore. (**e**) Detection of streptacidin in the absence (top) and presence (bottom) of an avidin group. (**f**) Minimum bulk concentrations of streptavidin, polyclonal anti-biotin Fab fragments, and monoclonal anti-biotin IgG antibodies required to observe at least 30–100 translocation events per second. (Controlling the translocation of proteins through nanopores with bioinspired fluid walls) Panels (**d**–**f**) are reproduced with permission from ref. [112]. © 2011 Springer Nature. (**g**) Frequency of translocation events for BSA and Impβ in unmodified nanopores and nanopores modified with Nup98 and Nup153. Reproduced from ref. [168]. © 2011 Springer Nature. (**h**) Schematic of DNA origami structures captured by solid-state nanopores. Red current traces represent traces without DNA origami structures. Upon introduction of DNA origami structures, a mild current decrease (blue) occurs, followed by a deeper, longer blockage (dark blue) after several seconds. This indicates stabilization within seconds, allowing the structure to remain stably in the pore until electrophoretic forces are removed. Introduction of HALO-HSTF produces ideal translocation events (green). Reproduced from ref. [181]. © 2024 American Chemical Society.

For instance, Liebes-Peer et al. embedded the catalytic triad (His, Glu, Ser) of acetylcholinesterase into a β-sheet-prone amphipathic peptide sequence, transforming weak binding signals to phosphoester bonds into a large-scale cooperative conformational shift from a disordered coil to a β-sheet [179]. This structural change enlarges the effective nanopore diameter, producing a strong and sustained current enhancement that amplifies the signal and enables specific recognition of organophosphorus molecules. Their experiments revealed two event types: frequent short events with large current increases and rarer longer events with smaller increases. Both types followed a Gaussian distribution, as shown in Figure 12a. In contrast, control experiments with unmodified nanopores (Figure 12b) or pores coated with a scrambled-sequence peptide (Figure 12c) showed no blockage signals, confirming that the modification imparts specific detection capability.

Yusko et al. incorporated biotinylated lipids with functional headgroups into a lipid bilayer (Figure 12d) [112], where these mobile ligands act as “baits” that concentrate targets near the pore entrance. This allows high-frequency translocation events even at picomolar concentrations (Figure 12e), without enriching non-target proteins. Different analytes can be distinguished based on their affinity for the displayed ligands: high-affinity proteins induce frequent events at low bulk concentrations, whereas low-affinity proteins require concentrations over 300 times higher to achieve comparable translocation frequencies (Figure 12f).

Kowalczyk et al. mimicked the nuclear pore complex by covalently immobilizing FG-nucleoporins onto solid-state nanopores [168]. The resulting FG-Nup barrier is permeable to nuclear transport receptor–bearing proteins (e.g., Impβ), allowing rapid transit (~2.5 ms), but strongly suppresses translocation of receptor-deficient proteins like BSA by orders of magnitude (Figure 12g). Joty et al. integrated DNA origami structures into solid-state nanopores to form hybrid nanopores (Figure 12h) [181]. The confined geometry forces target proteins (e.g., holo-hSTf) into closer interaction with the pore, leading to deeper current blockages, longer dwell times, and larger event areas. Together, these amplified parameters significantly improve the signal-to-noise ratio, thereby enhancing both detection sensitivity and specificity.

#### 3.4.5. Ionic Filtering Effects and the Physical Bandwidth Limit

In the preceding sections, we discussed suppressing noise through optimized electronic components (amplifiers, chip capacitors) to enhance measurement bandwidth. However, even with ideal, noise-free amplifiers, the physical characteristics of the nanopore system itself impose a fundamental limitation on achievable bandwidth. This constraint arises from the ion transport process itself, rather than the measurement electronics.

**Figure 13:**
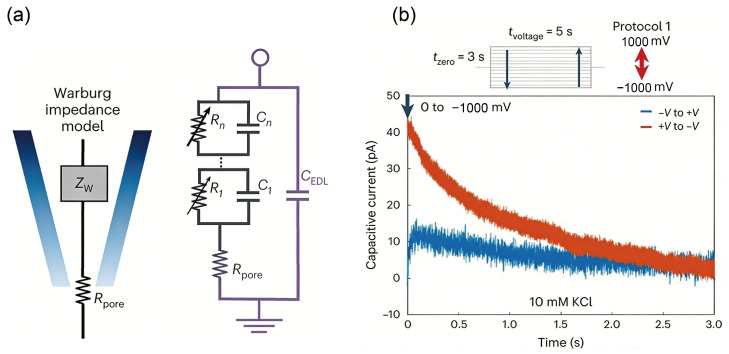
Warburg impedance model. (**a**) Proposed equivalent circuit model for a nanopipette that contains a Warburg element in series with the nanopore resistance. The Warburg element is modeled as a finite series of RC elements. (**b**) Negative capacitive current spike for the 0 V to −1000 mV voltage switch when protocol 1 was executed starting from −1000 mV versus starting at +1000 mV. Although the voltage pulse was the same, the history of the voltage pulses changes the capacitive current response. Panels (**a**,**b**) are reproduced with permission from ref. [182]. © 2025 Springer Nature.

The research by Farajpour et al. revealed this key mechanism [182]. They discovered that in nanopores, particularly those with asymmetric geometries like conical shapes, the attenuation of molecular transit events does not always stem from insufficient amplifier bandwidth. By ensuring the amplifier possessed adequate bandwidth, they observed that signal attenuation still occurred, demonstrating the existence of a pure ionic filtering effect. Since the Warburg element can be simplified to an RC element, Farajpour et al. proposed a new circuit model (Figure 13a). For singular Warburg elements, a finite number of RC components are required to simulate the slow decay of ion diffusion during the conical process. Each region of the taper possesses its own activation energy for generating and sustaining concentration polarization (CP). They attribute this ionic filtering effect to an element analogous to the “Warburg impedance” in electrochemistry, naming it “Warburg filtering.”

The physical origin of Warburg filtering lies in concentration polarization (CP) and its relaxation time. When a molecule (such as DNA) enters a nanopore, it physically displaces ions, instantly disrupting the ionic equilibrium between the pore interior and exterior, thereby creating a local ionic concentration gradient. To restore equilibrium, ions must redistribute through diffusion. This process is not instantaneous but requires a characteristic time τ (the relaxation time). The duration of this relaxation time depends on the pore geometry (particularly taper angle), solution properties, and the molecule itself. Figure 13b illustrates the establishment and dissipation of this CP, with two *V* = −1000 mV traces depicting a ~30 nm pore filled with 10 mM KCl (resistance components removed from each trace). By altering only the sequence of voltage pulses (starting from −1000 mV and increasing, versus starting from +1000 mV and decreasing), while keeping the pore, salt conditions, and voltage steps identical (i.e., the sole difference being the history of voltage pulses), Switching from positive to negative voltage causes the current to rise slowly from a low value, indicating the system exhibits “memory.” Conversely, switching from negative to positive voltage does not demonstrate this “memory” behavior.

In the frequency domain, this diffusion-dominated relaxation behavior manifests as a low-pass filter. High-frequency current changes—signals arising from rapid traversal or alterations in molecular internal structure—are smoothed or attenuated when attempting to drive ion redistribution, as ions cannot keep pace. Only signal components below a frequency of 1/τ can be transmitted with relatively little distortion. In other words, even if we someday possess an ideal amplifier with infinite bandwidth and zero noise, the inherent relaxation time of ion transport itself will act like an intrinsic low-pass filter, preventing us from directly resolving single bases through ionic currents. This forces us to look beyond ionic currents alone.

Lateral/vertical electron transport represents one of the most promising alternative approaches [183]. This strategy utilizes nanoelectrodes embedded within nanopores or constituting pore walls to directly measure tunneling currents or field-effect conductance during base passage. Since electron transport times in solids occur at the femtosecond scale, this signal remains entirely unaffected by slow ion diffusion processes in solution. Graphene nanoribbon (GNR) field-effect transistors exemplify this approach. Theoretical studies indicate that as DNA passes through, its charge modulates the GNR’s conductivity, generating a current signal 3 to 6 orders of magnitude higher than ionic currents. This enables single-base resolution at bandwidths around 10 MHz, significantly relaxing the requirements for measurement electronics.

The latest breakthrough stems from the application of vertical two-dimensional heterojunction diodes [184]. Researchers constructed a vertical van der Waals heterojunction and fabricated nanopores within it. When DNA translocations occurred, they achieved simultaneous detection of ionic currents and diode tunneling currents. Ionic currents continue to leverage their stable, easily measurable characteristics for precisely locating translocation events, while diode currents exhibit extreme sensitivity to local electric field perturbations at the base level, providing sequence-information-carrying signals free from Warburg attenuation. Notably, this work also discovered that the heterojunction potential can reduce the average DNA translocation speed by 2.3-fold through electrostatic interactions, simultaneously mitigating the “easy-to-pass-fast” issue.

#### 3.4.6. Machine Learning Overcomes Noise

In the past, we relied on conventional methods such as visual inspection of waveforms and scatter plots to differentiate distinct signals. However, distinguishing between characteristic signals and noise was often challenging when their features appeared similar, making them difficult to separate by eye or through basic analytical approaches. Today, machine learning (ML) offers a novel pathway. By employing models such as convolutional neural networks (CNNs), ML can automatically learn a set of high-dimensional, abstract, and noise-insensitive feature representations directly from raw current traces. The underlying principle is that during training, the model is exposed to vast amounts of noisy signal data. It learns to disregard high-frequency fluctuations that vary unpredictably across samples (noise), while focusing on stable, discriminative low-frequency patterns consistently present in signals from the same type of molecule. This approach is grounded in learning robust feature representations. Beyond that, ML leverages temporal sequence modeling by employing architectures such as recurrent neural networks (RNNs), long short-term memory networks (LSTMs), Transformers, or temporal convolutional networks (TCNs) to process an entire translocation event as a time series. This confers strong robustness against impulse noise and transient baseline jumps. ML methods are no longer merely a “promising” future direction but a fundamental component of the modern nanopore signal processing pipeline. Their role has evolved from simple pattern recognition to complex end-to-end basecalling. However, a critical analysis must consider the specific algorithms employed, the distinct problems they address, and their inherent limitations.

The problems tackled by ML can be broadly categorized into three levels of increasing complexity: (1) Event Detection and Feature Enhancement: At the most fundamental level, ML algorithms are used to identify true translocation events from noisy baselines and enhance features for downstream analysis. Classic approaches, often based on thresholding with digital filters (e.g., Bessel), have been augmented by more sophisticated techniques. Wavelet denoising, for example, transforms the signal into the frequency-time domain to effectively separate transient pulse-like signals from baseline noise, significantly improving the SNR for event detection [185]. Following detection, feature extraction (e.g., dwell time, mean current drop, event charge deficit) is typically performed. Unsupervised learning methods like k-means clustering are then commonly applied to this extracted feature space to classify events into distinct populations, such as differentiating unfolded, partially folded, and fully folded DNA translocations, or separating short DNA fragments by length [102]. These methods are interpretable and computationally efficient but are limited by the quality and information content of the hand-crafted features they rely on; (2) Deep Learning for End-to-End Basecalling: A paradigm shift occurred with the introduction of deep neural networks capable of directly converting raw electrical signals into nucleotide sequences, bypassing the need for intermediate event detection and feature extraction. The CHIRON model [186,187]. exemplifies this approach. It integrates a Convolutional Neural Network (CNN) with a Recurrent Neural Network (RNN) and a Connectionist Temporal Classification (CTC) decoder. The CNN acts as a feature learner, automatically identifying relevant local patterns (e.g., characteristic shapes of current fluctuations) from raw signal segments. The RNN (or more advanced versions like Long Short-Term Memory, LSTM) captures the long-range temporal dependencies between these features, modeling the context essential for accurate sequence decoding. Finally, the CTC decoder translates the RNN’s probability outputs into the final DNA sequence, elegantly handling the problem of variable alignment between signal segments and base positions. This end-to-end approach is now the industry standard (e.g., in Oxford Nanopore’s Guppy basecaller) and has proven vastly superior to earlier segmentation-based methods, particularly in handling homopolymer regions and noisy data; (3) Addressing Specific Biophysical Challenges: Beyond basecalling, deep learning is being deployed to tackle more nuanced biophysical challenges. For instance, the stochastic nature of DNA movement, especially the configurational entropy of ssDNA discussed in Section 3.2.6, leads to complex, non-uniform translocation speeds. Advanced architectures like Transformers, which utilize a self-attention mechanism, are particularly well-suited to model such long-range dependencies and variable-rate processes, potentially improving the accuracy of mapping signal fluctuations to specific positions on the DNA strand [188,189]. Furthermore, ML models are being developed to infer molecular identity or structural information directly from the complex signal “fingerprint”, moving beyond simple size discrimination.

Despite their power, ML methods have critical limitations. First, they are data-hungry, requiring vast, high-quality, and well-labeled datasets for training, which are not always available for novel analytes or experimental conditions. Second, they often function as “black boxes”, making it difficult to interpret the underlying physical or chemical features the model has learned to recognize, which can hinder scientific insight. Third, generalizability is a major concern; a model trained on data from one pore type, salt condition, or temperature may perform poorly when applied to different experimental setups. Finally, the computational cost of training and running large models can be prohibitive for real-time applications on portable devices. Therefore, the choice of ML approach must always balance predictive power with interpretability, resource constraints, and the specific nature of the problem.

## 4. Conclusions and Future Perspectives

Since Sanger established DNA sequencing in 1977, the technology has advanced through three generations. Third-generation platforms, such as those from Pacific Biosciences and Oxford Nanopore, are transforming genomics with long read lengths, direct sequencing, and real-time analysis. Solid-state nanopores, a key branch of this generation, show strong potential in genomics and diagnostics due to their mechanical stability, tunable pore size, and ease of integration.

The method works by detecting changes in ionic current as DNA passes through a nanoscale pore under an electric field. In theory, each base should produce a unique current signal. However, key challenges remain, including limited resolution, pore clogging, electrical noise, and insufficient sensitivity. These issues currently prevent reliable single-base resolution and wider use of the technology.

To overcome these obstacles, researchers have developed innovative strategies across multiple fronts. These include exploring ultrathin membrane materials to improve spatial resolution; modulating physical conditions, solution environments, nanopore geometry, surface modification, and bio-solid hybrid channels to regulate excessive DNA translocation speeds; reducing noise and enhancing the signal-to-noise ratio via material optimization, surface coating, passivation, and device architecture design; alleviating pore clogging through surface modifications and pulsed electric fields; and boosting sensitivity and specificity by means of surface chemical functionalization, biomimetic designs, and integration with DNA origami structures.

Although solid-state nanopore technology is still in its development and optimization phase, its potential for single-molecule detection, long-read sequencing, and real-time diagnostics is undeniable. Building on prior achievements and identified challenges, future research should focus on several key directions. In terms of material innovation, it is essential to continue exploring novel two-dimensional materials and composites that combine ultrathin geometry, low noise, and excellent biocompatibility. For instance, MXene materials show great promise in this regard [190,191]. Like graphene, MXenes are atomically thin, but their surfaces are rich in hydrophilic functional groups such as hydroxyl and fluorine, making them inherently hydrophilic. Compared to other 2D materials such as MoS_2_ and h-BN, which are hydrophilic but either semiconducting or insulating, MXenes offer high electrical conductivity similar to metals. This metallic conductivity allows MXene films to serve as efficient electrodes under an applied voltage, supporting novel sensing mechanisms. Furthermore, the abundant surface functional groups on MXenes provide ideal sites for in situ covalent modification. This enables specific recognition elements, such as aptamers or peptide chains, to be grafted onto or within the nanopores, thereby supporting highly selective molecular detection.

Another important direction is the integration and scalability of nanopore technology, which involves advancing the combination of nanopore chips with readout circuitry to enable high-throughput, low-cost, and portable sequencing platforms. Furthermore, intelligent signal analysis leveraging artificial intelligence and deep learning can help develop more accurate current-to-base mapping models, thereby improving sequence recognition precision. Additionally, machine learning and neural networks can be applied to predict ideal molecular structures for surface functionalization. Inspired by the transformative impact of AlphaFold in structural biology [192], similar computational approaches can be used to design optimal modification molecules based on desired performance traits. This strategy could significantly enhance the effectiveness of nanopore chemical modifications and accelerate the development and application of solid-state nanopore technologies.

While solid-state nanopore research has produced a wealth of laboratory demonstrations, the transition to practical applications will likely be driven by three key areas that directly address the fundamental physical limits outlined in this review.

First, hybrid biological–solid-state nanopores represent the most mature path toward practical sequencing. By embedding biological pores (e.g., MspA, phi29) into robust solid-state membranes, this approach combines the atomic precision of protein pores with the mechanical stability of solid-state devices. The use of enzymatic motor control (e.g., phi29 DNA polymerase) to achieve stepwise, ratcheted translocation has already proven successful in commercial platforms (Oxford Nanopore Technologies), offering a clear trajectory from laboratory validation to real-world deployment.

Then, direct electronic readout modalities offer a transformative alternative to ionic current sensing. Graphene nanoribbon field-effect transistors and vertical 2D heterojunction diodes generate signals orders of magnitude larger than ionic current and are inherently immune to the Warburg filtering effects that fundamentally limit ionic current bandwidth. The simultaneous detection of ionic and diode currents in heterojunctions provides a powerful dual-channel readout, positioning this as the most exciting frontier for achieving true single-base resolution.

Active spatial control techniques such as nanopore-based scanning ion conductance spectroscopy depart fundamentally from stochastic translocation. By tethering DNA molecules and actively scanning them through the pore, these methods offer deterministic control over translocation speed and position, enabling applications requiring ultra-high precision—such as mapping protein-binding sites or detecting rare epigenetic modifications—even if they do not become high-throughput sequencing platforms.

Across all three directions, machine learning will serve as the essential bridge between raw signal and biological insight, with roles ranging from wavelet denoising to end-to-end basecalling. Ultimately, the translation of solid-state nanopores from laboratory curiosity to practical tool will depend not on incremental improvements, but on the strategic convergence of biological precision, electronic readout, and computational analysis.

## Data Availability

No new data were created or analyzed in this study.

## Abbreviations

The following abbreviations are used in this manuscript:

CVD	Chemical Vapor Deposition
ALD	Atomic Layer Deposition
EOF	Electroosmotic Flow
PSD	Power Spectral Density
CMOS	Complementary Metal-Oxide-Semiconductor
CNP	CMOS-Integrated Nanopore Platform
His	Histidine
Glu	Glutamic
Ser	Serine
RCA	Rolling Circle Amplification
Mxene	2D Transition Metal Carbide/Nitride
CNN	Convolutional Neural Network
FC	Fully Connected
LSTM	Long Short-term Memory
RNN	Recurrent Neural Network
